# Utilizing PBF-LB/M AlSI10Mg alloy post-processed via KOBO-extrusion and subsequent cold drawing to obtain high-strength wire

**DOI:** 10.1038/s41598-025-14980-3

**Published:** 2025-08-23

**Authors:** P. Snopiński, A. Appiah, K. Matus, Ł. Kuczek, K. Żaba, M. Balcerzak, J. Hajnyš

**Affiliations:** 1 Department of Engineering Materials and Biomaterials, Silesian University of Technology, 18A Konarskiego Street, 44-100 Gliwice, Poland; 2 Materials Research Laboratory, Faculty of Mechanical Engineering, Silesian University of Technology, 18A Konarskiego Street, 44-100 Gliwice, Poland; 3 Department of Metal Working and Physical Metallurgy of Non-Ferrous Metals, AGH University of Science and Technology, Al. Adama Mickiewcza 30, 30-059 Cracow, Poland; 4 Faculty of Mechanical Engineering, VSB-TU Ostrava, 17. Listopadu 2172/15, 708 00 Ostrava, Czech Republic

**Keywords:** AlSi10Mg, Cold-drawing, KOBO extrusion, Microstructure characterization, Post-processing treatment, Mechanical properties, Metals and alloys

## Abstract

This study investigates the efficacy of a multi-stage thermo-mechanical processing route, comprising KOBO extrusion followed by cold drawing, for enhancing the mechanical properties of AlSi10Mg alloy produced via by powder bed fusion laser beam melting (PBF-LB/M). The objective was to develop high-strength wires from this additively manufactured alloy. The microstructural evolution throughout the processing sequence was characterized using scanning electron microscopy (SEM), electron backscatter diffraction (EBSD), and transmission electron microscopy (TEM). KOBO extrusion transformed the initial as-built microstructure into a fine-grained, composite-like structure, characterized by a refined α-Al matrix with homogeneously distributed, fragmented Si particles. This resulted in a compelling combination of mechanical properties, including an ultimate tensile strength (UTS) of 324 MPa and a total elongation of approximately 10%. Subsequent cold drawing induced significant strain hardening and further grain refinement, achieving an average grain size of − 700 nm. This raised the UTS to 401 MPa, though it was accompanied by a substantial reduction in ductility to − 0.6%. These findings demonstrate that the synergistic combination of KOBO extrusion and cold drawing is a promising pathway for fabricating high-strength AlSi10Mg wires from PBF-LB/M -processed feedstock.

**Supplementary Information:**

The online version contains supplementary material available at 10.1038/s41598-025-14980-3.

## Introduction

In metal additive manufacturing (AM), a complex interplay of metallurgical phenomena significantly determines the properties and quality of printed parts^1–3^. The process begins by heating the metal powder to its melting point in the printing chamber, followed by rapid solidification. These unique fabrication conditions yield microstructures different from those of conventional manufacturing processes. Specifically, AM produces unique non-equilibrium cellular microstructures that are crucial for the superior mechanical properties of as-fabricated components^4^. However, despite the advantages provided by AM technology, notable drawbacks persist. The main challenges currently faced in PBF-LB/M include formation of < 100 > columnar grains^4,5^, as well as issues like porosity^6^, residual stresses^7^ and lack of fusion defects^8^. Columnar grains introduce anisotropy, influencing mechanical properties, while porosity affects both quasistatic mechanical characteristics and corrosion resistance^9^. Residual stresses and lack of fusion defects further degrade the material’s mechanical integrity, leading to reduced strength, increased brittleness, and a higher likelihood of crack formation during service^10,11^. These issues can make AM components unsuitable for certain structural applications.

To address these challenges, post-processing treatments are widely applied to modify the microstructure and improve mechanical properties. For instance, thermomechanical treatments such as hot extrusion or friction stir processing are common for conventional Al-Si casting alloys^12,13^. In contrast, the PBF-LB/M Al-Si counterparts often undergo isostatic pressing^14,15^, T6 heat treatment^16^ or other post-processing treatments^17,18^, which result in a significant transformation of the microstructure. These treatments typically convert the original cellular structure into a composite-like structure with uniformly distributed isolated Si particles. However, such transformations often lead to a deterioration of mechanical properties, including reduced strength and ductility^19–21^. This has driven interest in hybrid approaches that combine AM with traditional forming processes to retain and enhance material performance.

One such hybrid method is Ampliforge™, developed by Alcoa in 2015, which integrates PBF-LB/M with forging to mitigate defects and improve material properties^22^ Hot forming has also been explored as a post-processing strategy for PBF-LB/M parts^23^, offering the potential to refine grains through recrystallization, address anisotropy, and enhance mechanical performance^23^.

While studies on post-processing methods like hot and cold forming have shown promise^24–28^, the literature on multistage post-processing of PBF-LB/M alloys remains limited. The demand for high-strength materials with optimized microstructures underscores the need for deeper exploration of plastic deformation mechanisms and dislocation behavior in PBF-LB/M Al-Si alloys.

The KOBO extrusion method used in this study is a unique post-processing technique that offers several advantages over conventional extrusion. The cyclic deformation path in KOBO extrusion induces dynamic recrystallization at lower temperatures, fostering grain refinement while preserving ductility—a challenge for conventional extrusion^29,30^. This reduces extrusion force and eliminates the need for annealing, making the process more energy-efficient and cost-effective. Additionally, the high-frequency changes in deformation allow the cold forming of materials that are difficult to deform, such as magnesium alloys^31^.

One of the most notable benefits of the KOBO method is its ability to produce ultra fine-grained materials. This microstructural refinement significantly enhances the mechanical properties of the material, such as yield strength and toughness, while maintaining or even improving ductility^32,33^. For instance, research has shown that magnesium alloys processed via KOBO extrusion exhibit significant enhancements in both grain refinement and mechanical performance when compared to their conventionally processed counterparts^34^. In some cases, the KOBO method has even enabled the production of nanostructured materials with grain sizes of 20–60 nm^35^. These refined microstructures serve as an excellent starting point for subsequent post-processing, such as cold drawing or rolling, without compromising the material’s integrity, allowing additional refinement of both microstructure and properties.

This work investigates the effect of cold drawing on the microstructure and mechanical properties of a PBF-LB/M AlSi10Mg alloy post-processed using the KOBO method. The study compares three distinct microstructures: the as-fabricated PBF-LB/M condition, the KOBO-extruded condition, and the KOBO-extruded and cold-drawn condition. Microstructural characterization was conducted using optical, scanning electron, and transmission electron microscopy, correlating the observations with mechanical performance. Finally, the relationship between microstructure and mechanical properties is discussed, and a strengthening model is proposed for the cold-drawn samples.

The proposed multi-stage post-processing strategy holds significant promise for aerospace and automotive sectors, where high strength-to-weight ratios are critical, and the use of recycled powder aligns with sustainable manufacturing goals.

## Methodology

The AlSi10Mg sample analyzed in this study was fabricated using a TruPrint 1000 PBF-LB/M system. The detailed alloy composition is provided in Table 1. The manufacturing process was carried out using the following optimized process parameters:

- laser power of 175 W,
- scanning speed of 1400 mm/s,
- layer thickness of 20 μm,
- rotation angle of 67°,
- vertical printing direction,
- protective gas Ar.
- Extrusion ratio (λ): 225 (from ϕ60 to ϕ4 mm), resulting in a true strain of 5.42.
- The final specimen had a circular cross-section.

**Table 1:** The AlSi10Mg alloy powder chemical composition in wt%.

Al	Mg	Si	Ti	Cu	Fe
87.8	0.5	10.5	0.15	0.15	0.09

The cylindrical sample, measuring 50 mm in length and 60 mm in diameter, underwent post-processing via the KOBO (Korbel-Bochniak) method, see Fig. S1. This Severe Plastic Deformation (SPD) technique is particularly effective for challenging metallic materials, as its core principle involves the cyclic, oscillatory rotation of the extrusion die superimposed on the punch’s forward movement during the extrusion process^36^. The material was extruded in a single step under the following conditions:

- Punch speed: 0.2 mm/s,
- Die oscillation frequency: 5 Hz,
- Die oscillation angle: 8°,

The cold drawing stage was performed on a drawing bench machine operating at a constant speed of 20 mm/s. To reduce friction and ensure a smooth surface finish, the wire surface was coated with MoS₂ lubricant throughout the drawing cycles. The detailed processing parameters are summarized in Table 2. A schematic illustration of the entire processing route is provided in Fig. S1 (Supplementary file).

**Table 2:** Process parameters applied for cold drawing of KOBO-processed sample.

Diameter of drawn wire (mm)	No. of pass	Total RA (%)	Effective drawing strain
4.0	–	0	0.00
3.78	1	10	0.11
3.66	2	16	0.18
3.56	3	20	0.23
3.44	4	26	0.30
3.29	5	32	0.39
3.14	6	38	0.48
3.0	7	43	0.57
2.86	8	48	0.67

The reduction in area (RA) per pass during the wire drawing test was determined using the following formula:

1 $$\:RA=\frac{{A}_{0}-{A}_{f}}{{A}_{0}}\times\:100\left(\%\right)$$

where *A*_o_ represents the initial cross-sectional area and *A*_f_ corresponds to the final cross-sectional area. The effective drawing strain was estimated using the following formula:

2 $$\:\epsilon\:=ln\frac{{A}_{0}}{A}$$

Where *ε* refers to the effective drawing strain.

Following each second drawing cycle, a 60 cm long sample was taken for the purpose of microstructural and mechanical properties characterization. Consequently, in this study, the analysis includes samples deformed to effective strains of 0.18, 0.3, 0.48, and 0.67.

Samples were ground with 800- and 1200-grit abrasive papers and polished with 3.0 and 1.0 μm diamond pastes. Final polishing used a 60 nm Al_2_O_3_ colloidal suspension for 60 min. X-ray diffraction (XRD) analysis was conducted to calculate the dislocation density. The diffraction measurements spanned the range from 20° to 120°, with a step size of 0.01° and a counting interval of 5 s per step. We used a PANalytical X’Pert Pro diffraction system equipped with a cobalt anode (Kα = 1.789 Å).

The secondary electron images were captured at an accelerating voltage of 20 kV. EBSD analysis was performed using an accelerating voltage of 20 kV and step sizes ranging from 0.25 to 0.08 μm to characterize the grain structure. The 5° grain tolerance angle (GTA) and 9-pixel grain size threshold were selected to accurately delineate grain boundaries and capture fine-scale microstructural features typical of post-deformation samples.

A Focused Ion Beam (FIB) was utilized to prepare electron-transparent foils for TEM and high-resolution TEM (HRTEM) analyses. The milling process employed Ga ions, executed in several sequential steps, to achieve a final foil thickness of approximately 120 nm. The TEM lamellas were extracted and oriented along the direction of extrusion.

TEM and HRTEM examinations were performed using a Titan 80–300 FEI scanning transmission electron microscope (S/TEM), operating at an accelerating voltage of 300 kV. To analyze electron diffraction patterns, Gatan DigitalMicrograph software (version 2.32.888.0) and CrysTBox (Crystallographic Toolbox, version 1.1)^37^ software was used.

The Vickers microhardness (HV) examinations were performed on the cross-sectional plane of the as-built, KOBO-processed, and cold-drawn samples using a microhardness tester from Future-Tech company. At each indentation point, a load of 100 gf was applied, with a dwell time of 15 s.

Tensile tests on the extruded wires (diameter ≤ 4 mm) were performed at room temperature using a Zwick Z20 electromechanical universal tester. The tests followed the constant rate (0.001 s^−1^) specified in ASTM A370 (Annex A4) for wire products. No specimen preparation was needed; the full cross-section of the wires was tested.

## Results

**Fig. 1:**
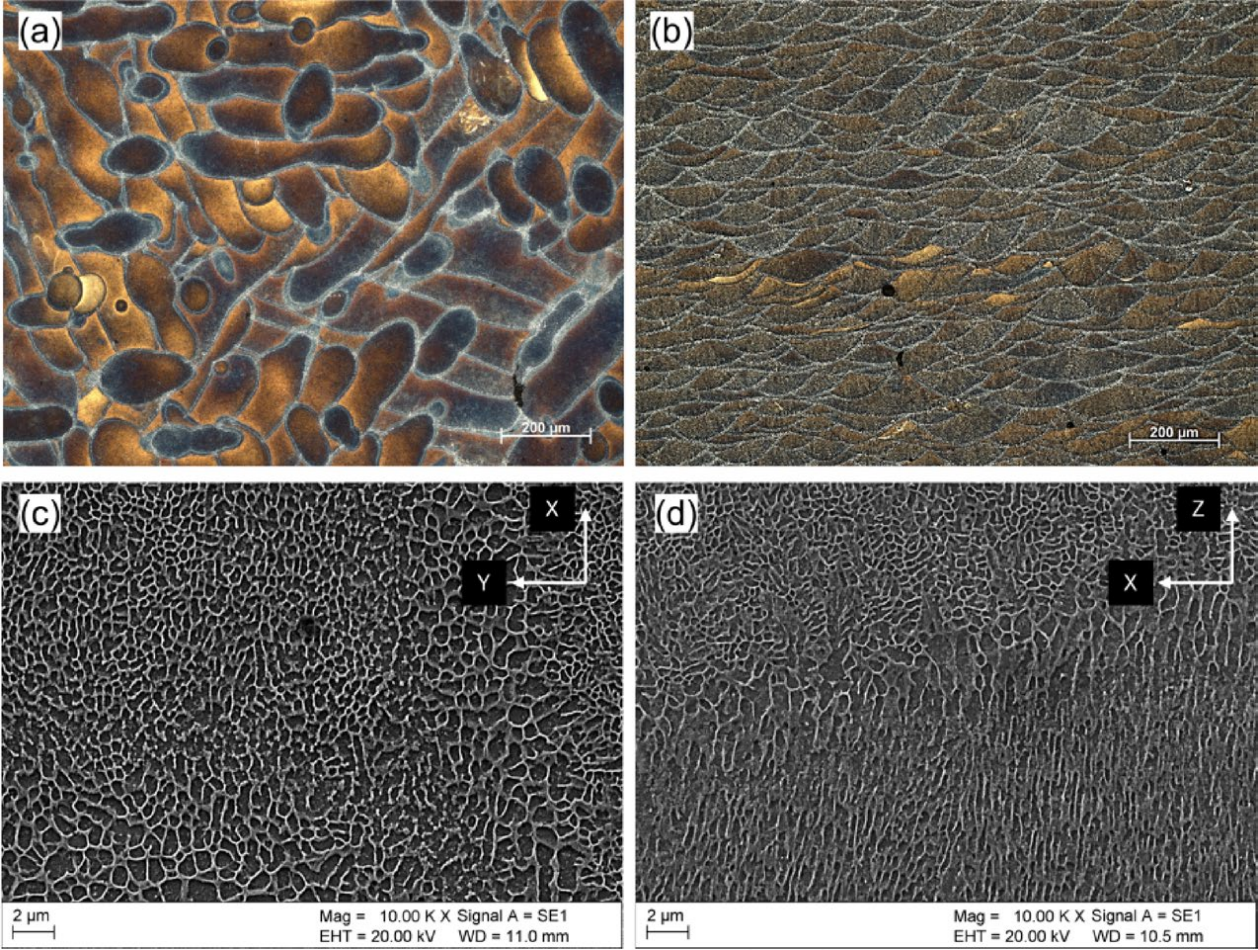
Microstructures of the AlSi10Mg alloy in the as-built condition: (**a**) Optical Microscopy (OM) image of the X-Y plane, (**b**) OM image of the X-Z plane, (**c**) Scanning Electron Microscopy (SEM) image of the X-Y plane, and (**d**) SEM image of the X-Z plane. Note that the Z-axis represents the shorter side of the image.

Figure 1 presents the microstructures of the as-built sample. For the X-Y plane (Fig. 1a), chemical etching exposes discontinuous laser scan traces. The boundaries of these traces etch more prominently, leading to their brighter appearance in the optical microscopy (OM) image. This enhanced susceptibility to chemical etching in these specific regions is caused by the presence of fibrous (divorced) eutectic Si particles.

The X-Z plane (Fig. 1b) microstructure is composed of characteristic fish-scale (semicircular) patterns. These patterns arise from the periodic overlapping of adjacent melt pools during laser scanning, directly reflecting the alternating heat input and cooling cycles fundamental to the fabrication process.

Figure 1c and d present the SEM micrographs of the microstructure on the transverse (X-Y) and longitudinal (X-Z) planes, respectively. On the XY plane, the microstructure is characterized by a network of fine Al-Si eutectic cells. The formation of this cellular structure is attributed to the rapid solidification and high cooling rates inherent to the fabrication process^38^. Upon closer inspection, this plane can be classified into three distinct regions based on variations in the morphology and size of the Al-Si eutectic phase:

- The melt pool (MP) fine region – with relatively fine Al-Si cells,
- MP coarse region – featuring coarser Al-Si cells,
- Heat-affected zone – featuring ruptured eutectic Al-Si network.

The morphology of the Si eutectic is dependent on the observed section view. When viewed in the plane parallel to the build direction (X-Z plane), the Al-Si eutectic exhibits a columnar morphology, aligning itself with the thermal gradient. Consistent with observations from the X-Y plane, the Al-Si eutectics within the melt pool (MP) fine zone are larger compared to those in the MP coarse zone, which is evident in Fig. 1d.

**Fig. 2:**
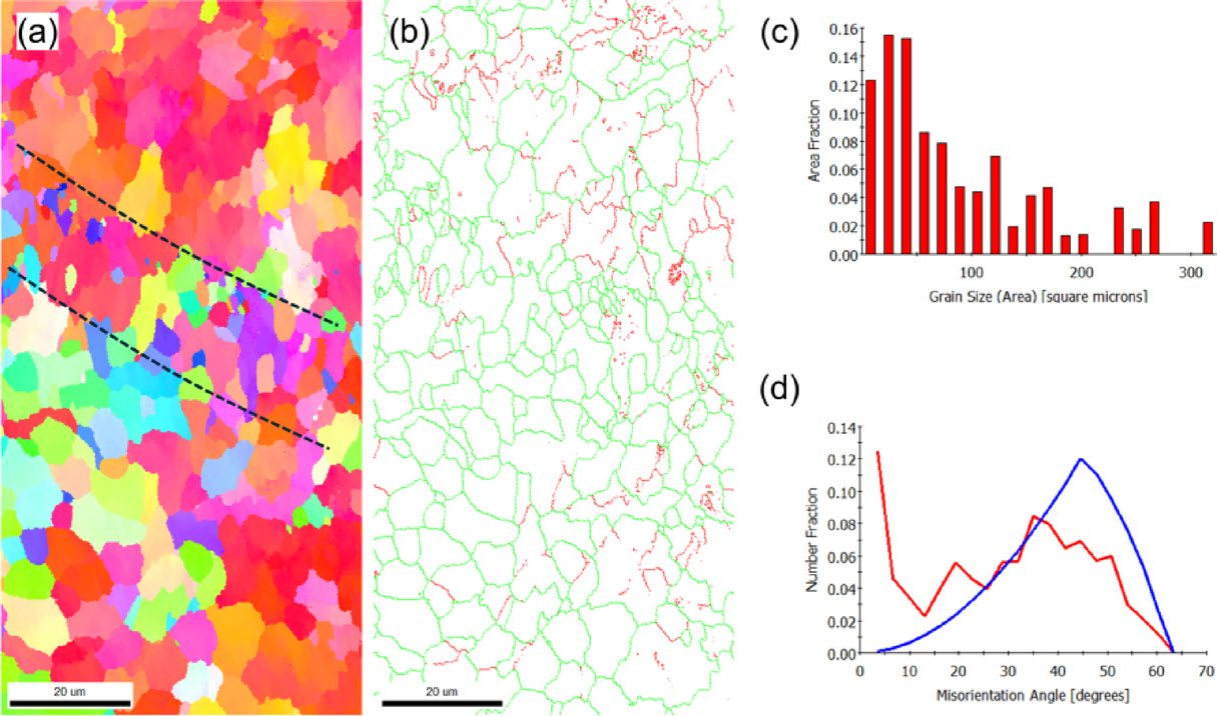
EBSD results of the as-built (X-Y plane) sample: (**a**) IPF-Z map, (**b**) grain boundary map showing low-angle grain boundaries (red) and high-angle grain boundaries (green), (**c**) grain size distribution histogram, and (**d**) misorientation angle histogram. The blue line represents the theoretical random distribution of grain misorientation.

**Fig. 3:**
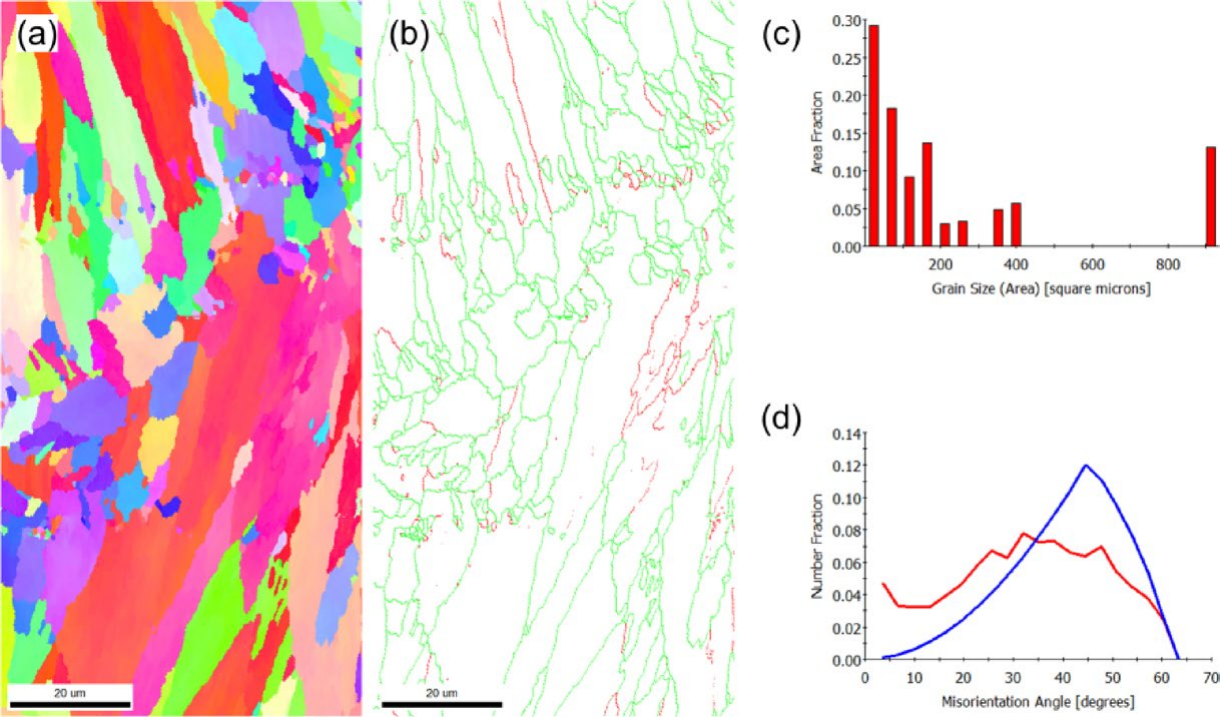
EBSD results of the as-built (X-Z plane) sample: (**a**) IPF-Z map, (**b**) grain boundary map showing low-angle grain boundaries (red) and high-angle grain boundaries (green), (**c**) grain size distribution histogram, and (**d**) misorientation angle histogram.

EEBSD was employed to characterize the grain structure of the as-built sample on the X-Y and X-Z planes (Figs. 2 and 3, respectively). The inverse pole figure (IPF) map of the X-Y plane, shown in Fig. 2a, reveals a bimodal grain distribution. This structure consists of fine grains concentrated at the laser scan trace boundaries (marked by dashed lines) and coarse, equiaxed grains dominating the interior of the scan paths. The statistical evaluation yields an average grain area of 22 μm^2^ (Fig. 2c), and a mean linear intercept length of 3.5 μm (X-direction) and 3.4 μm (Y-direction). The close agreement between the intercept lengths confirms the predominantly equiaxed nature of the grain morphology.

The grain boundary map (Fig. 2b) and the misorientation distribution histogram (Fig. 2d) reveal that low-angle grain boundaries (LAGBs), particularly those with misorientation angles in the range of 2°–5°, are predominant. This observation aligns with findings from Liu et al.^39^, who reported that LAGBs in PBF-LB/M parts are typically composed of dense dislocation structures. These structures accommodate the significant residual thermal stress caused by the repeated melting and cooling cycles during the fabrication process.

In the X-Z plane, which aligns with the build direction, columnar grains are prominently visible, as shown in Fig. 3a. The IPF image predominantly features red hues, which visually confirm the alignment of grains with the < 001 > crystallographic direction attributed to the easy growth direction in FCC metals^40^. Quantitative analysis reveals that these columnar grains, reaching lengths of up to 100 μm, have an average grain area of 19 μm^2^ (Fig. 3c). The anisotropic nature of this structure is further confirmed by the mean linear intercept lengths, which measured 2.8 μm along the X-axis and 3.5 μm along the Y-axis (build direction).

The grain boundary map (Fig. 3b) and the corresponding misorientation distribution histogram (Fig. 3d) reveal a slightly higher proportion of high-angle grain boundaries (HAGBs) in the X-Z plane compared to the XY plane. Specifically, HAGBs account for 85%, while LAGBs constitute the remaining 15% of the boundary fraction.

**Fig. 4:**
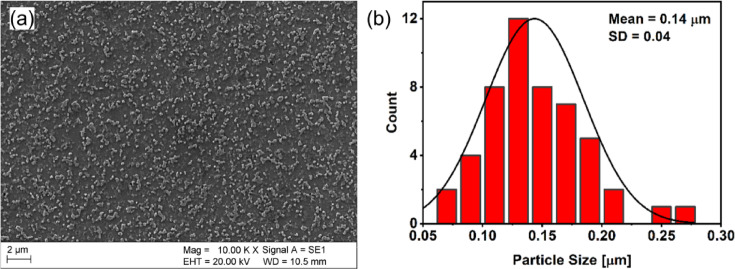
(**a**) SEM image of the KOBO-processed sample and (**b**) particle size distribution measurement.

Figure 4a illustrates the microstructural evolution of the AlSi10Mg alloy after KOBO extrusion post-processing. The SEM image reveals a significant refinement of the Al-Si cellular structure, with Si particles more uniformly distributed within the aluminum matrix (Supplementary Fig. S2). Examining the particle size distribution in Fig. 4b, it becomes evident that most Si particles fall within the range of 0.05–0.20 μm. The average size of the silicon particles measured is 0.14 ± 0.04 μm.

**Fig. 5:**
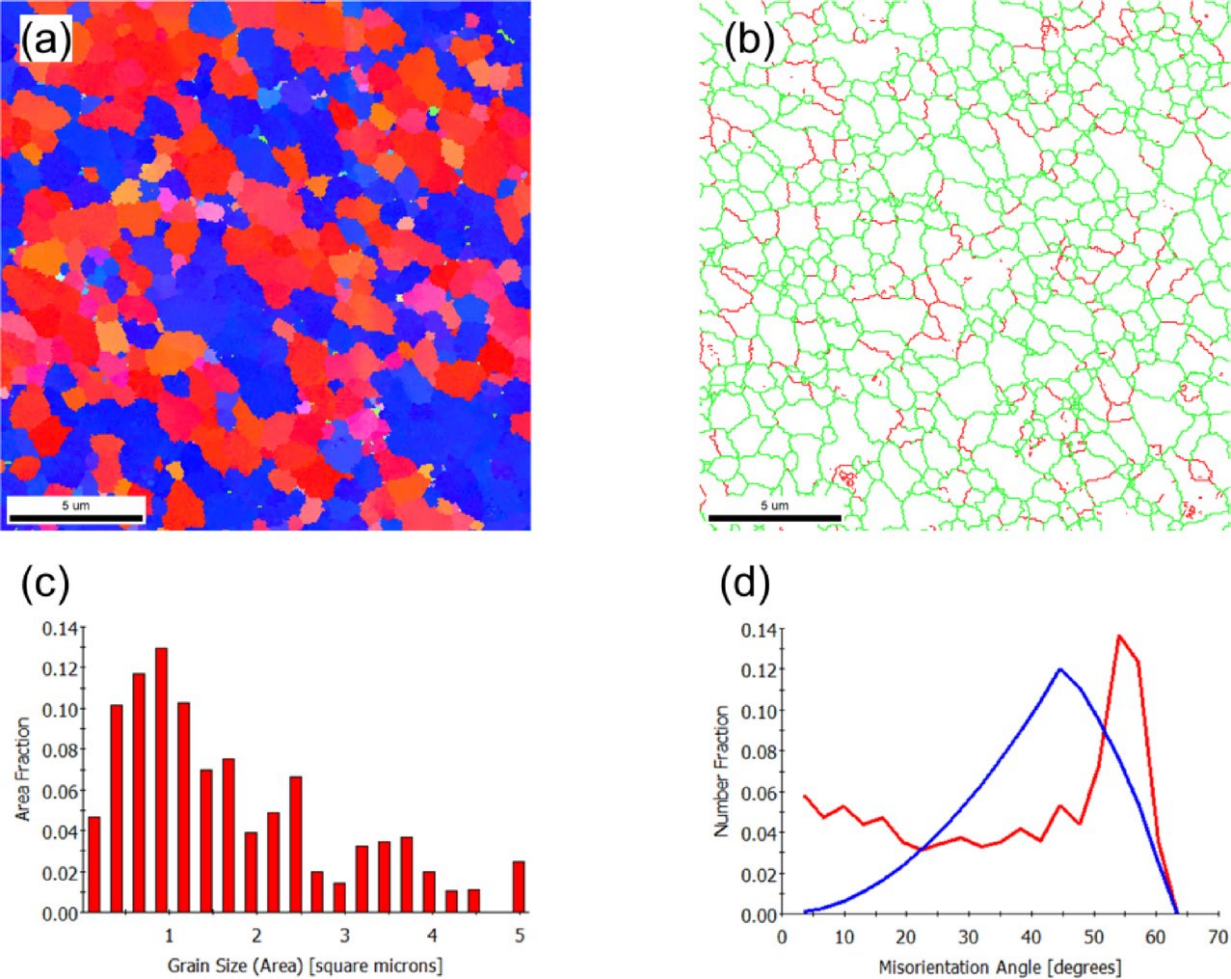
EBSD results of the KOBO-processed AlSi10Mg alloy sample: (**a**) IPF-Z map, (**b**) grain boundary map showing low-angle grain boundaries (red) and high-angle grain boundaries (green), (**c**) grain size distribution histogram, and (**d**) misorientation angle histogram.

Figure 5 shows the grain structure of the AlSi10Mg alloy after KOBO extrusion, as characterized by EBSD. The inverse pole figure (IPF-Z) map (Fig. 5a) confirms that severe plastic deformation induced significant grain refinement. The microstructure is composed of equiaxed grains with an average area of approximately 0.8 μm^2^, corresponding to an equivalent grain size of ~ 0.9 μm. This uniform refinement, driven by dynamic recrystallization (DRX)^32^, is further evidenced by the grain size distribution histogram (Fig. 5c), which shows a narrow distribution with the majority of grains falling between 0.2 and 1.8 μm^2^.

Figure 5b displays the grain boundary map, while Fig. 5d presents the corresponding grain boundary misorientation histogram. Experimental data indicate that LAGBs constitute approximately 19% of the total grain boundary fraction, with HAGBs accounting for the remaining 81%.

**Fig. 6:**
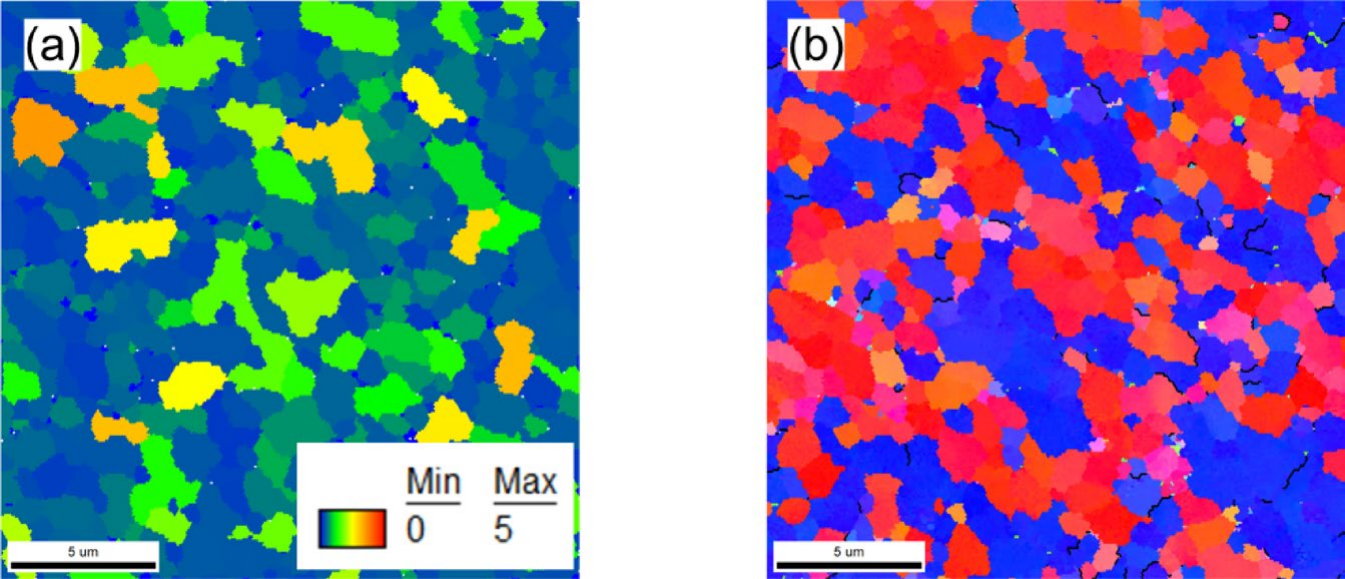
EBSD analysis of the KOBO-processed AlSi10Mg alloy. (**a**) Grain Orientation Spread (GOS) map, with grains possessing a GOS value below 1° colored blue to highlight the dynamically recrystallized (DRX) fraction of 86%. (**b**) Inverse Pole Figure (IPF-Z) map showing the crystallographic orientation. The black lines delineate Σ3 Coincidence Site Lattice (CSL) boundaries, indicating the presence of annealing twins.

According to the data presented in Fig. 6, a significant fraction of recrystallized grains (with GOS < 1°) are delineated by grain boundaries with misorientation angles ranging between 55° and 65°. Such grains are typically identified as recrystallization twins, and according to Fig. 6b, these grains predominantly have the misorientation axis oriented near the < 111 > direction, i.e. 60/<111 > twins. Within the recrystallized fraction, approximately 1.8% of the grain boundaries correspond to special coincidence site lattice (CSL) ∑3 twin boundaries (black boundaries). The prevalence of these twins is attributed to the strong fiber texture, which not only orients grains favorably for active deformation twinning but also statistically increases the frequency of twin-like boundaries as a purely geometrical consequence^41,42^.

**Fig. 7:**
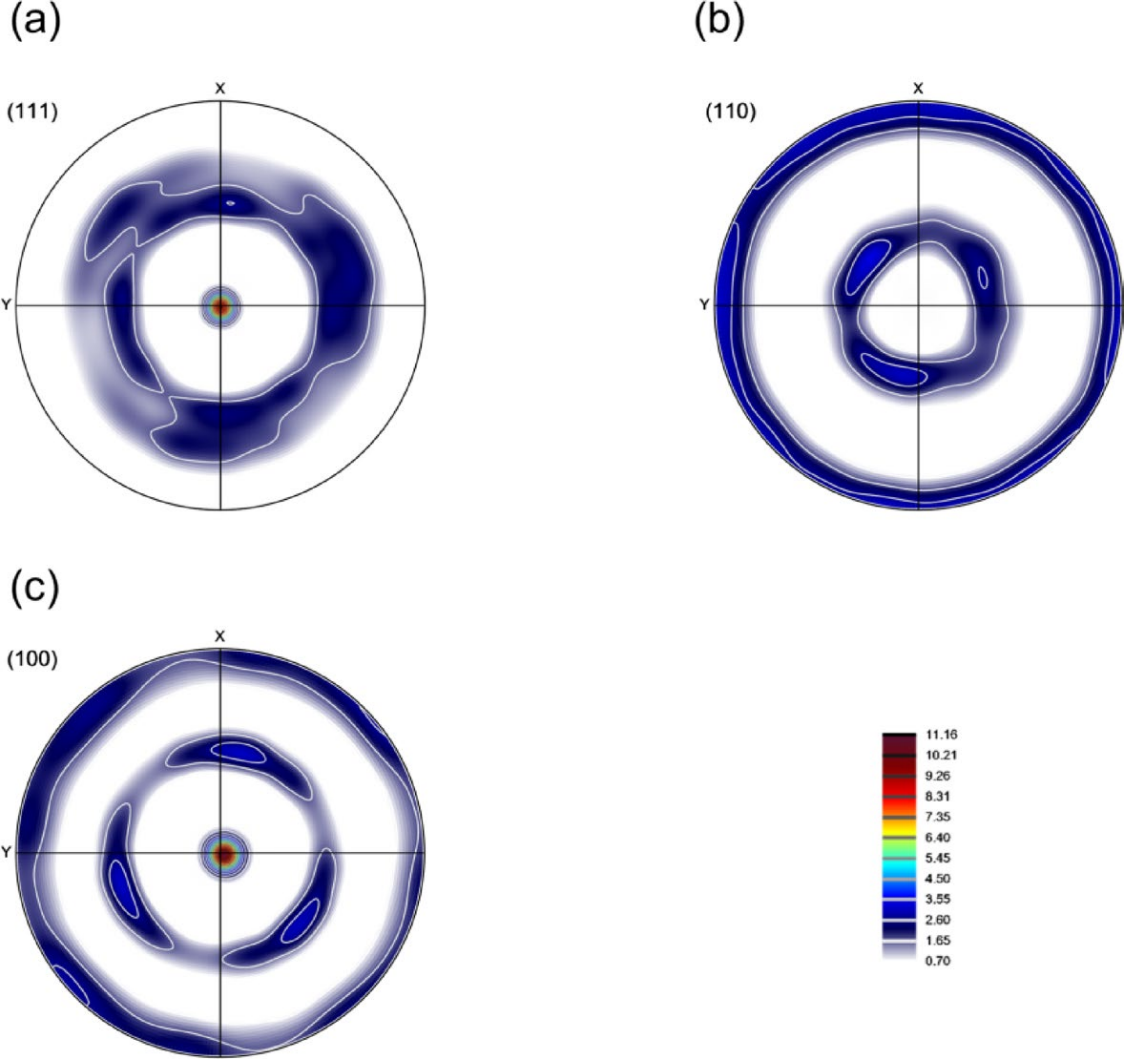
Pole figures of the KOBO-processed AlSi10Mg alloy sample. *Note*: x corresponds to TD, and y corresponds to ND.

Figure 7 presents the pole figures from the texture analysis, revealing a distinct double fiber texture characteristic of hot-extruded face-centered cubic (FCC) materials. The formation of this crystallographic texture is a direct consequence of the near plane-strain deformation conditions imposed during the KOBO extrusion process. Under these conditions, the crystallographic orientations of the grains tend to align along two primary fibers within the Euler space. These are identified as:

- The α-fiber, which encompasses a range of orientations with a common < 110 > direction parallel to the normal direction (ND). This fiber typically extends from the Goss orientation, defined by the Miller indices {110}<001>, to the Brass orientation {110}<112>.
- The β-fiber, which extends from the from Copper orientation {112}<111 > through S orientation {123}<634>.

**Fig. 8:**
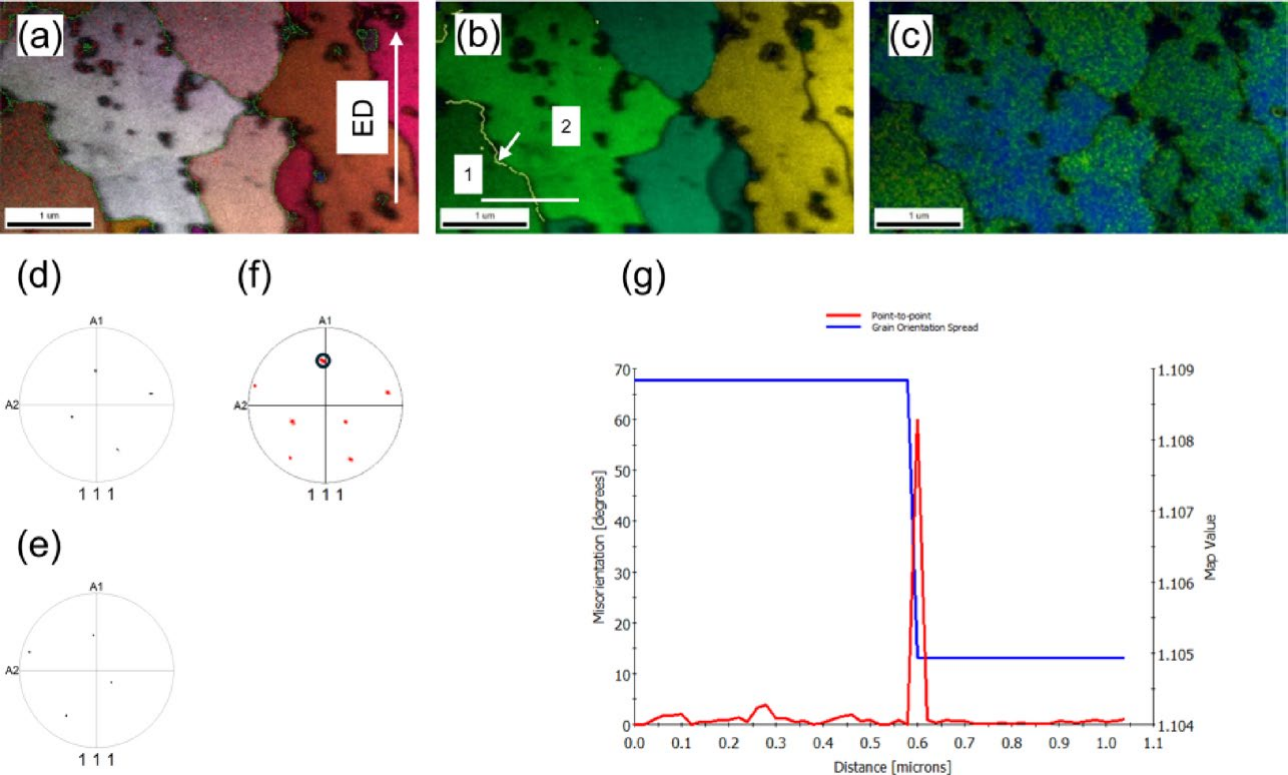
TKD experiment results: (**a**) band contrast map with superimposed IPF map, (**b**) GOS map, (**c**) kernel average misorientation (KAM) map, (**d**) pole figure for grain (1) in (**b**), (**e**) pole figure for grain (2) in (**b**), and (**f**) combined pole figure. Pole figures reveal twin relationship with (111) twinning plane. (**g**) Point-to-point misorientation and GOS values along the white line in (**a**). GOS and KAM are color-coded from blue (0°–1°) to red (4°–5°).

Further insights into the grain structure of the KOBO-processed sample are provided by high-resolution transmission Kikuchi diffraction (TKD) analysis, Fig. 8. The IPF-Z map (Fig. 8a) reveals several grains elongated in the direction of extrusion, primarily separated by high-angle boundaries (green lines in the Fig. 8a), and this observation aligns with the EBSD results presented above.

To analyze strain distribution within individual grains, the grain orientation spread (GOS) was calculated (Fig. 8b). Most grains exhibit GOS values below 2°, indicating minimal strain accumulation within these regions. However, strain heterogeneities become more apparent in the kernel average misorientation (KAM) map (Fig. 8c). Elevated KAM values are observed near dynamically recrystallized (DRX) grains, which appear to grow through a bulging mechanism (indicated by white arrows). These DRX grains emerge from areas with low geometrically necessary dislocation (GND) density, expanding into regions with higher GND density. This phenomenon suggests that grain boundaries migrate along strain gradients, with boundaries moving toward more distorted regions. Consequently, strain-induced boundary migration (SIBM) leads to corrugated boundary profiles, as described in earlier studies^43^.

The twin relationship between specific grains is further elucidated in the pole figures (Fig. 8d and e), which demonstrate the crystallographic twin orientation between grains labeled (1) and (2). The orientation scattering, shown within the black circle in Fig. 8f, results primarily from rotation around the {111} twinning plane. Additionally, the accumulated misorientation profile along the white line confirms the presence of a 60° twin boundary, as depicted in Fig. 8g.

**Fig. 9:**
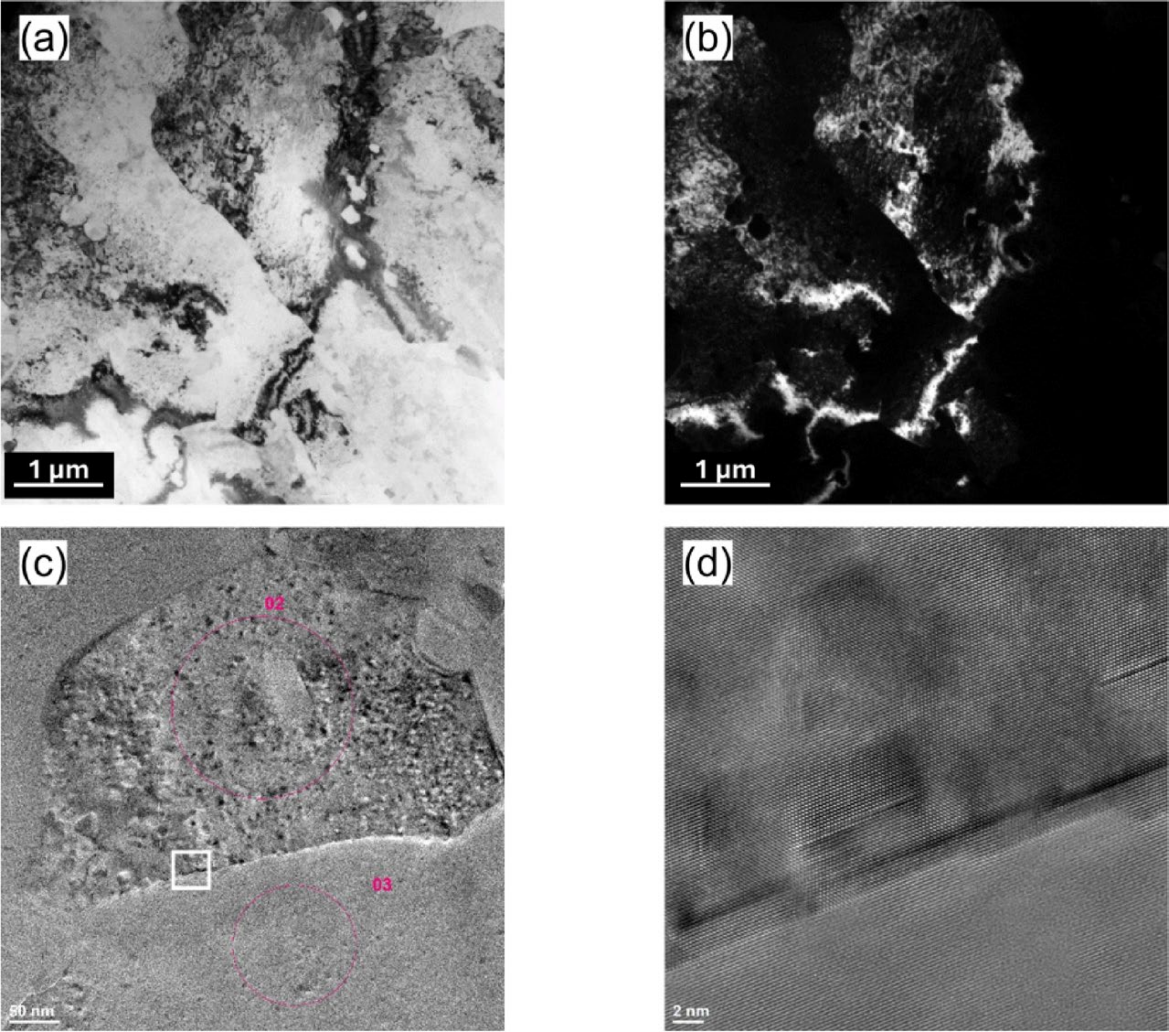
TEM observations of the KOBO-processed sample: (**a**) bright-field TEM image, (**b**) dark-field TEM image showing dislocation structures within grains, (**c**) magnified TEM image of the grain boundary area, and (**d**) HRTEM image corresponding to the white square in (**c**).

TEM has been used to obtain additional information about the grain structure of the KOBO-processed sample. Figure 9a and b reveal submicrometer grains, which mainly exhibit an elongated shape and have sizes ranging between 0.7 μm and 2.5 μm. Also, in the area analyzed, the grains are heavily strained through some dislocation tangles. These areas of tangled dislocations obviously represent non-DRX grains.

The high-resolution TEM image reveals twin boundaries (TBs) have a unique faceted structure composed of longer coherent (111) and shorter semicoherent nanofacets (see Fig. 9cand d). It is also interesting to note that the semi-coherent nanofacets are not exactly at 90° to the coherent facet, as might be expected, but are slightly inclined. Based on the observed twin’s morphology, we identify it as an annealing twin since it lacks the lenticular shape characteristic of a deformation twin^44^.

**Fig. 10:**
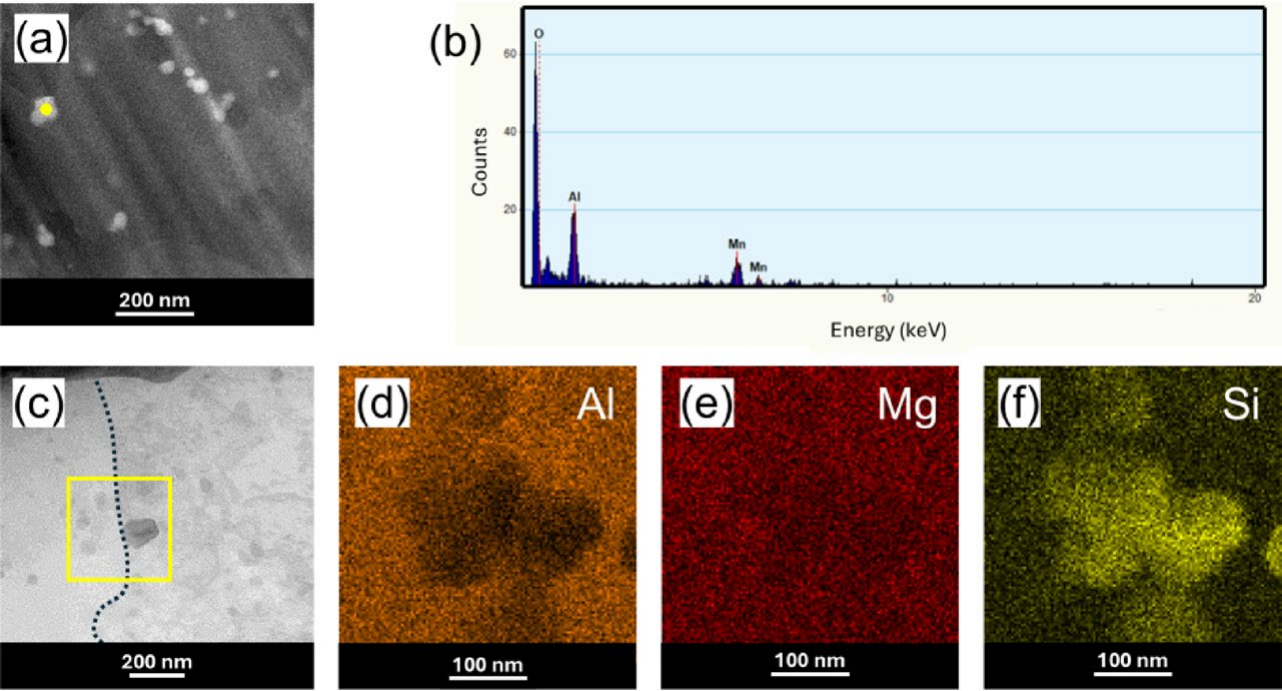
EDX and elemental mapping results: (**a**) STEM-HAADF image, (**b**) EDX spectrum for point (1) in (**a**), (**c**) STEM image, (**d**) Al element map, (**e**) Mg element map, and (**f**) Si element map. EDS maps were taken from the yellow square shown in (**c**).

To closely observe the distribution of the secondary phases, we used the STEM mode. The STEM-HAADF image (Fig. 10a) reveals numerous nanoscale precipitates distributed in the alloy matrix, which are identified as oxides according to the EDS in Fig. 10b. The STEM-EDX chemical maps in Fig. 10d–f detail the concentration of Al, Mg, and Si elements within the yellow box area in Fig. 10c and confirm that the Si particles tend to agglomerate along the grain boundaries (dotted line).

**Fig. 11:**
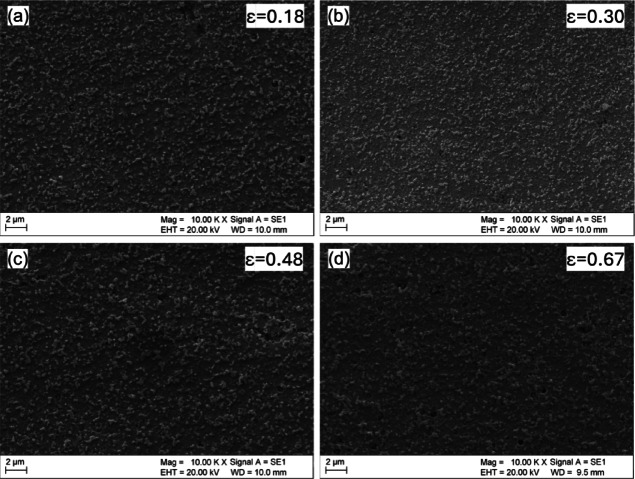
SEM images of the KOBO-processed AlSi10Mg sample after cold drawing: (**a**) effective strain 0.18, (**b**) effective strain 0.30, (**c**) effective strain 0.48, and (**d**) effective strain 0.67.

Figure 11a–d shows the evolution of the second phases during the cold drawing process. The Si precipitates are uniformly sized and appear as individual spherical particles as well as agglomerates, randomly distributed throughout the alloy matrix. The size of the Si precipitates remains consistent with those observed in the KOBO-processed sample, indicating that the cold drawing process has little to no impact on their refinement.

**Fig. 12:**
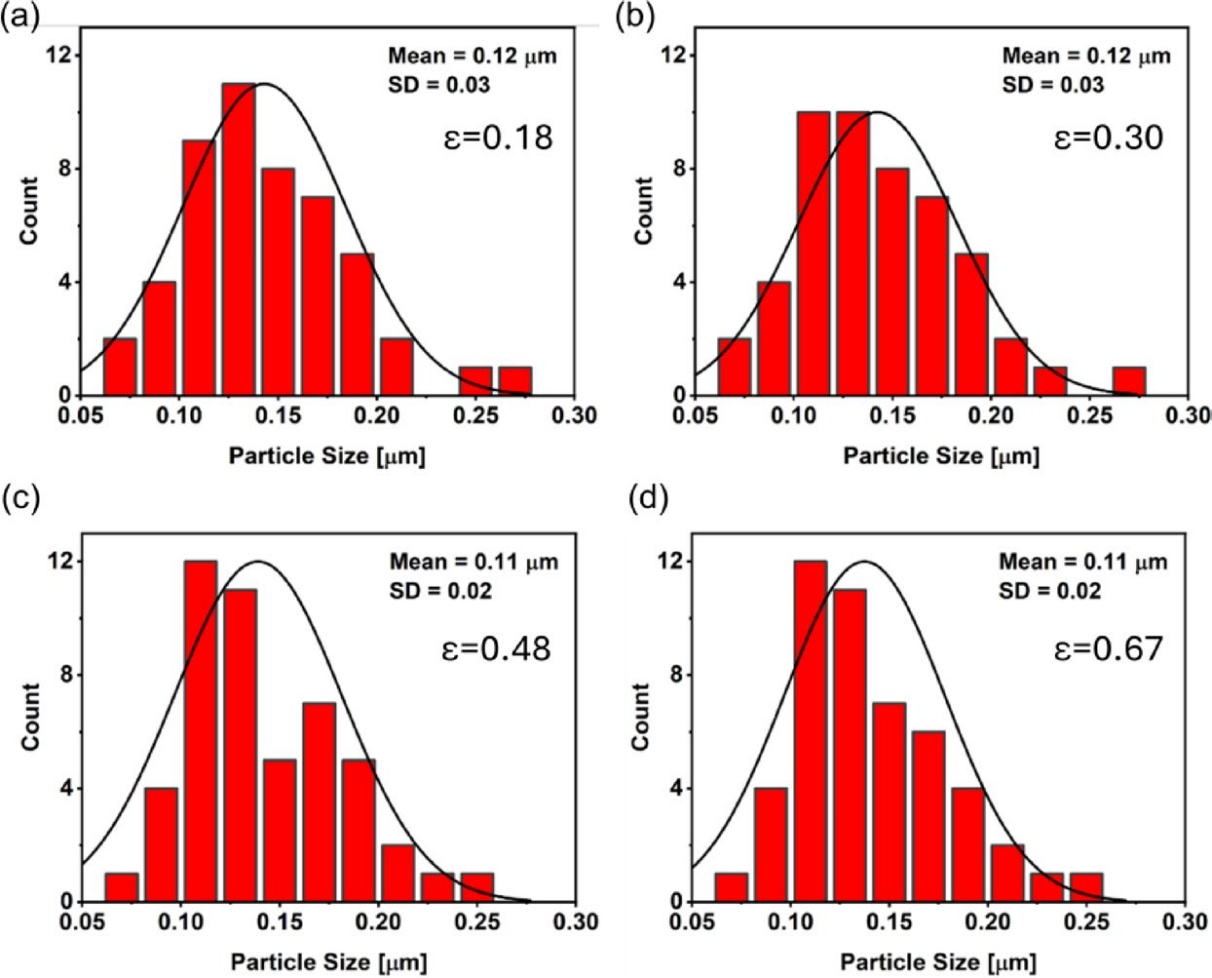
Particle size distribution histograms of the KOBO-processed AlSi10Mg sample after cold drawing: (**a**) effective strain 0.18, (**b**) effective strain 0.30, (**c**) effective strain 0.48, and (**d**) effective strain 0.67. The subtle difference in standard deviation suggests a more uniform particle size distribution at higher strain levels, which might be attributed to the more homogeneous deformation of the material during the cold drawing process.

Figure 12 shows histograms of the particle size distribution following cold drawing. In the sample subjected to an effective strain of 0.18, the average particle size is 0.12 ± 0.03 μm (Fig. 12a). Similarly, at an effective strain of 0.30, the particle size remains 0.12 ± 0.03 μm (Fig. 12b). For higher effective strains of 0.48 (Fig. 12c) and 0.67 (Fig. 12d), the particle size stabilizes at 0.11 ± 0.02 μm. The distribution is dominated by particles ranging from 0.08 to 0.12 μm, with an overall average particle size of 0.11 μm, confirming a minor refinement due to cold drawing when compared to the KOBO-processed sample.

**Fig. 13:**
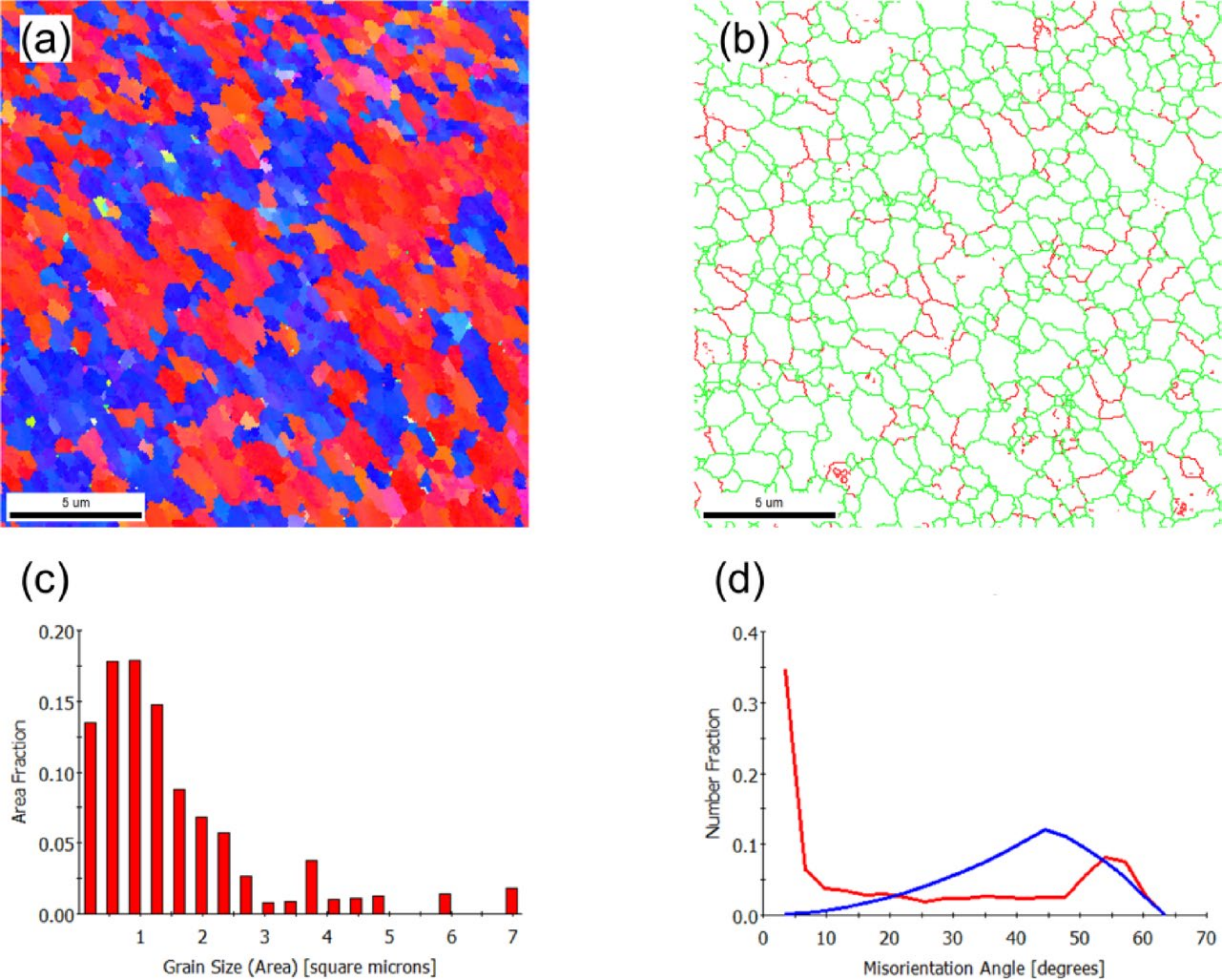
EBSD results of the KOBO-processed AlSi10Mg alloy sample after cold drawing (effective strain 0.48): (**a**) IPF-Z map, (**b**) grain boundary map showing low-angle grain boundaries (red) and high-angle grain boundaries (green), (**c**) grain size distribution histogram, and (**d**) misorientation angle histogram.

Figure 13 illustrates the effect of cold drawing (effective drawing strain of 0.48) on the grain structure evolution. The cold-drawn sample exhibits a finer grain structure compared to the KOBO-processed counterpart, Fig. 13a. As can be seen in Fig. 13c, the grain area distribution ranges from 0.1 to 3 μm, with an average grain area of approximately 0.47 μm^2^.

Figure 13b shows the grain boundary map, while Fig. 13d shows the misorientation angle histogram. The misorientation angles exhibit a bimodal distribution with peaks at approximately 10° and 55°. Additionally, the population of low-angle grain boundaries (LAGBs) is relatively higher due to the increased density of DRV substructures (subgrains). Experimental data show that LAGBs constitute about 48% of the total grain boundary fraction, while high-angle grain boundaries (HAGBs) to the remaining 52% of the boundary fraction. The twin boundary peak is significantly smaller than in the KOBO-processed sample, suggesting that most twin boundaries have lost their twin characteristics during cold deformation. This phenomenon is mainly due to concurrent deformation accompanied by crystal rotations, which alter the grain orientation.

**Fig. 14:**
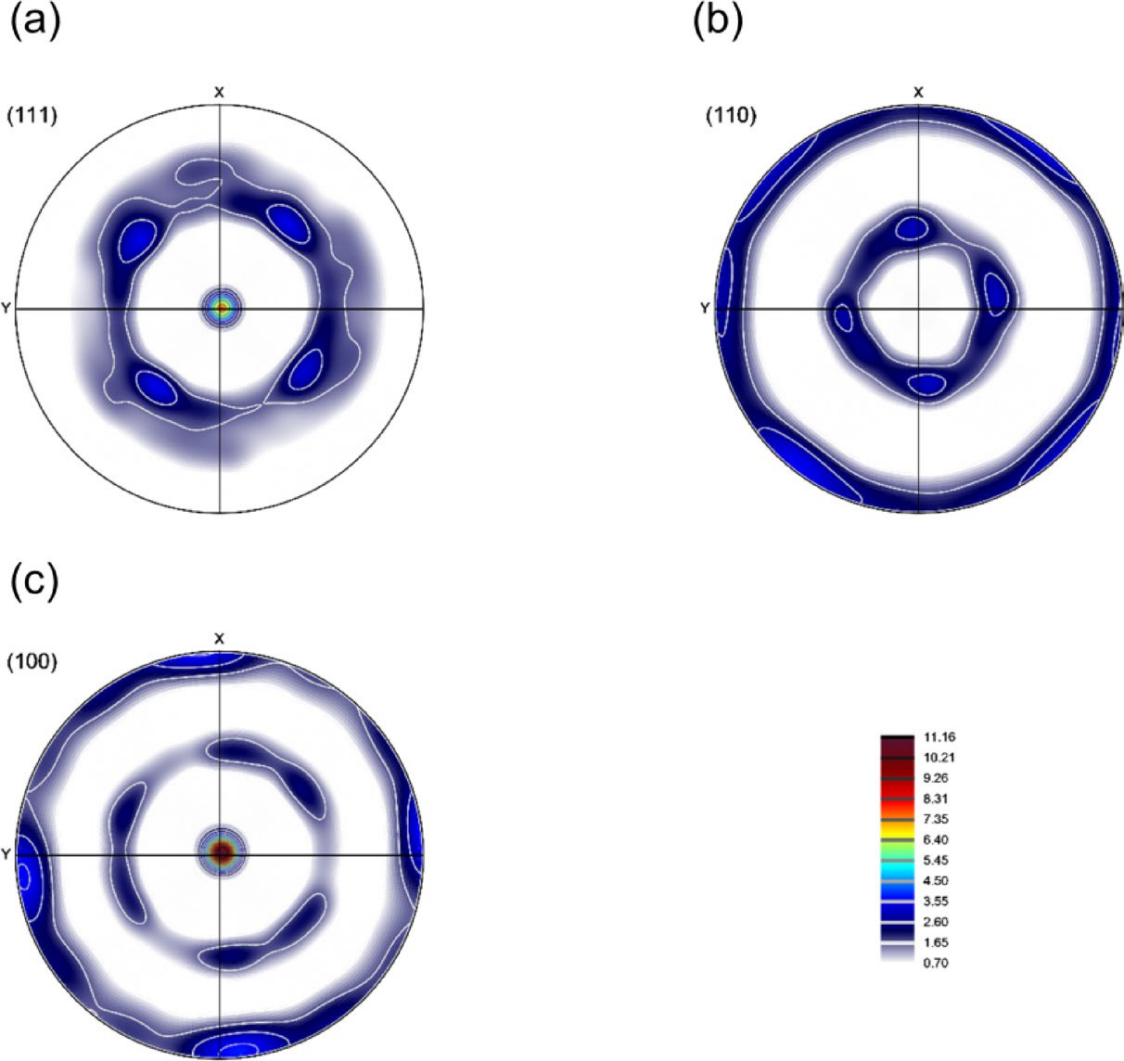
Pole figures of the KOBO-processed AlSi10Mg alloy sample after cold drawing (effective strain 48%). *Note*: no EBSD data rotation was applied; x corresponds to TD, and y corresponds to ND.

Figure 14 presents the pole figures of the cold-drawn sample, revealing a pronounced double-fiber texture characteristic of this deformation process. The texture is defined by the preferential alignment of the < 111 > and < 100 > crystallographic directions with the drawing axis. A significant intensification of the < 100 > fiber component is particularly evident, a typical outcome of extensive plastic deformation induced by cold drawing in face-centered cubic metals^45^.

**Fig. 15:**
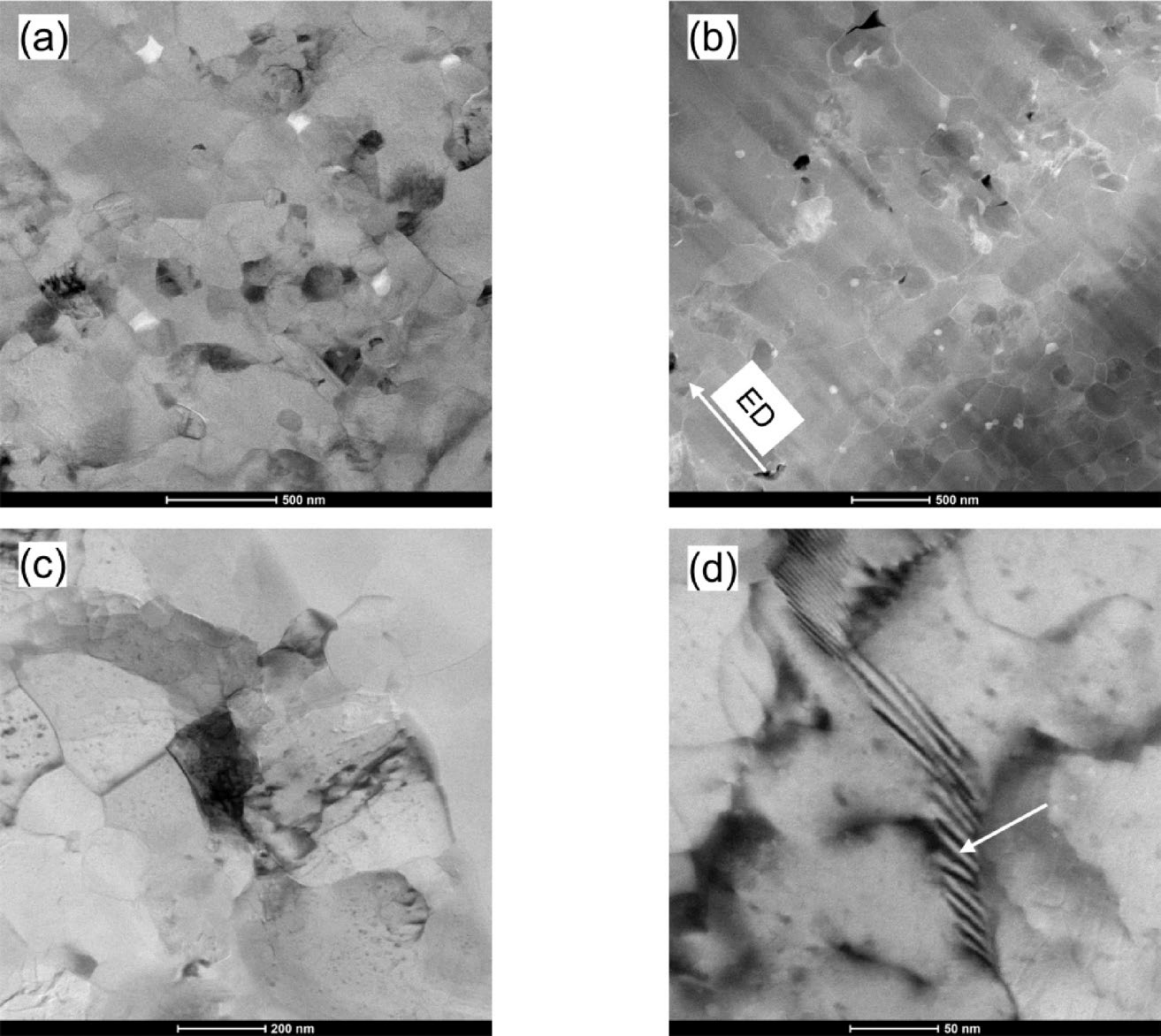
TEM Analysis of Cold-Drawn KOBO-Processed AlSi10Mg (ϵ=0.48): (**a**) low-magnification STEM image showing the grain structure, (**b**) corresponding STEM-HAADF image, (**c**) high-magnification bright-field STEM image showing dislocation arrangement within the grains, and (**d**) high-magnification STEM image showing dislocation walls (red arrow).

The evolution of microstructure is further investigated using TEM. Figure 15 confirms the significant structural changes caused by cold drawing. The low-magnification STEM image shows numerous subgrains separated by low-angle dislocation structures (Fig. 15a). Most grains are larger than 500 nm; however, there are also grains smaller than 300 nm. This measured average grain size of 733 ± 41 nm is consistent with the results of the EBSD analysis.

The STEM-HAADF image (Fig. 15b) reveals several voids sized about 30 to 120 nm, highlighted by white arrows. These voids result from interface decohesion and Si particle fragmentation, indicating localized and inhomogeneous deformation. A closer look at the HAADF image reveals that cracks in the Si particles mainly grow in the direction of applied stress. Voids initiate from particle fractures during plastic deformation because hard Si particles limit elastic and plastic deformation, causing local stress concentrations.

The higher magnification STEM images offer greater insight into the alloy microstructure, as shown in Fig. 15c and d. Both images reveal high dislocation entanglement and dense dislocation walls (DDWs), formed to minimize the total energy state. This microstructure is characteristic of aluminum alloys, where the dominant softening mechanism is dynamic recovery^46^.

**Fig. 16:**
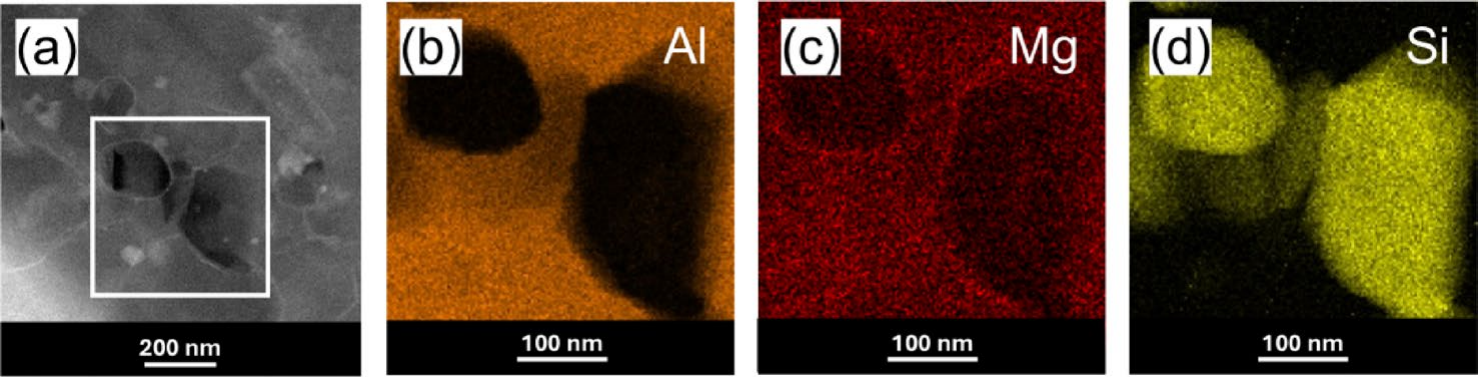
Elemental maps of cold-drawn (48% effective strain) KOBO-processed AlSi10Mg: (**a**) STEM image, (**b**) Al map, (**c**) Mg map, and (**d**) Si map. EDS maps correspond to the white square in this figure (**a**).

The STEM-EDS mapping (Fig. 16) confirms the chemical composition of numerous precipitates visible in the STEM images. It also supports the earlier assumptions that the precipitates remained unrefined during the cold drawing process. Specifically, the mapping reveals Si precipitates ranging from approximately 150 to 200 nm in size, consistent with those observed in the KOBO-processed sample (Fig. 11).

**Fig. 17:**
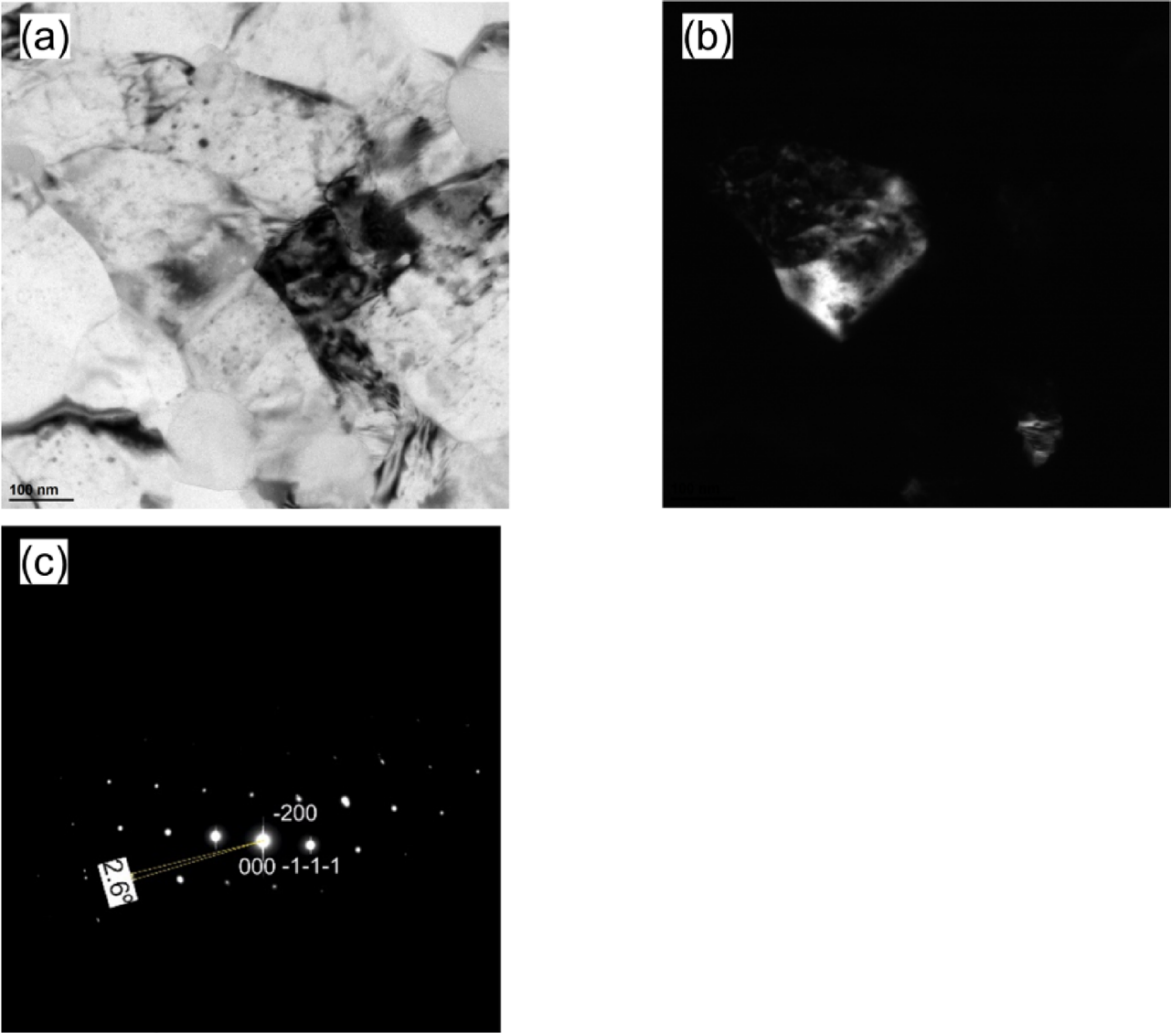
TEM observations of the KOBO-processed AlSi10Mg alloy sample after cold drawing (effective strain 48%): (**a**) bright-field TEM image showing the subgrain structure, (**b**) dark-field TEM image taken from the − 1-1-1 diffraction spot, and (**c**) selected area diffraction pattern (ZA = 0–11).

Figure 17 shows the BF-TEM and DF-TEM images along with the corresponding selected area diffraction pattern (SAED). In SAED we see two diffraction spots far away from the transmission spot, Fig. 17c. The angle measured between the two spots is approximately 2.6°, indicating that DDWs (that are confined by low angle boundaries (< 1°)) are rearranged and annihilated in some areas, creating subgrain boundaries with a higher misorientation angle compared to DDWs^47^.

**Fig. 18:**
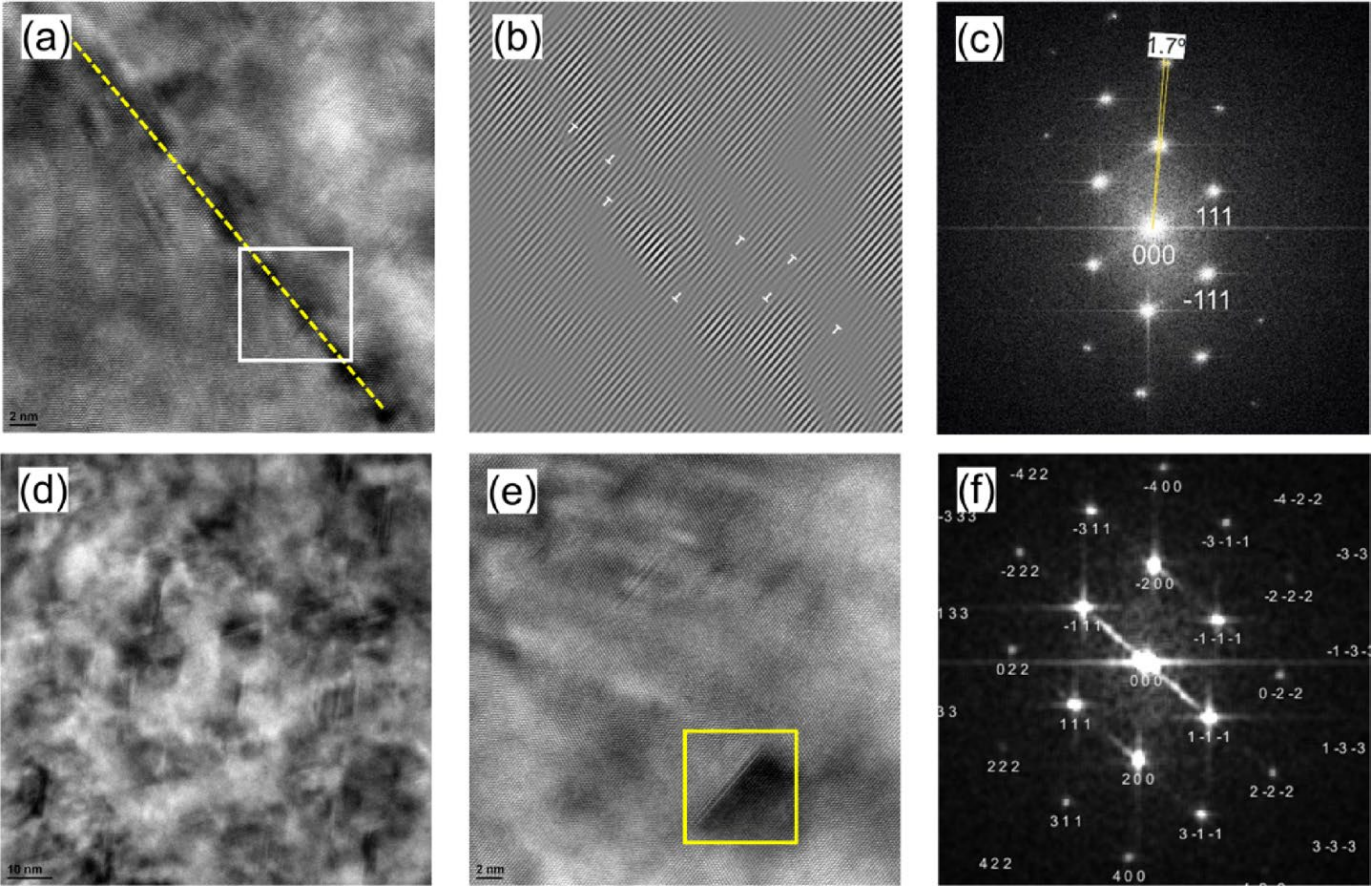
HRTEM images of the KOBO-processed AlSi10Mg alloy sample after cold drawing (effective strain 0.48): (**a**) HRTEM image of the grain boundary area, (**b**) IFFT image of the white box in (**a**) revealing multiple dislocations at the boundary, (**c**) FFT of (**a**), (**d**) HRTEM image showing a dense stacking fault network in the aluminum matrix, (**e**) high-magnification HRTEM image, and (**f**) FFT of the yellow box in (**e**) documenting stacking faults (SFs).

The HRTEM images in Fig. 18a and b align with the low-magnification TEM images, displaying a low-angle grain boundary (marked by the yellow dotted line) formed by the accumulation of high-density dislocations. The corresponding fast Fourier transform (FFT) reveals a misorientation between the two grains of about 1.7 degrees, confirming the LAGB (Fig. 18c).

A magnified HRTEM image (Fig. 18d and e) shows a dense stacking fault network extending along multiple {111} planes in the FCC grains, as can be seen from Fig. 18f. These stacking faults exhibit strong interactions with each other, indicating that twinning may be an additional deformation mechanism of the FCC Al phase. Overall, the results suggest a multiple grain refinement mechanism involving the dense dislocation walls, nanotwins and stacking faults formation.

**Fig. 19:**
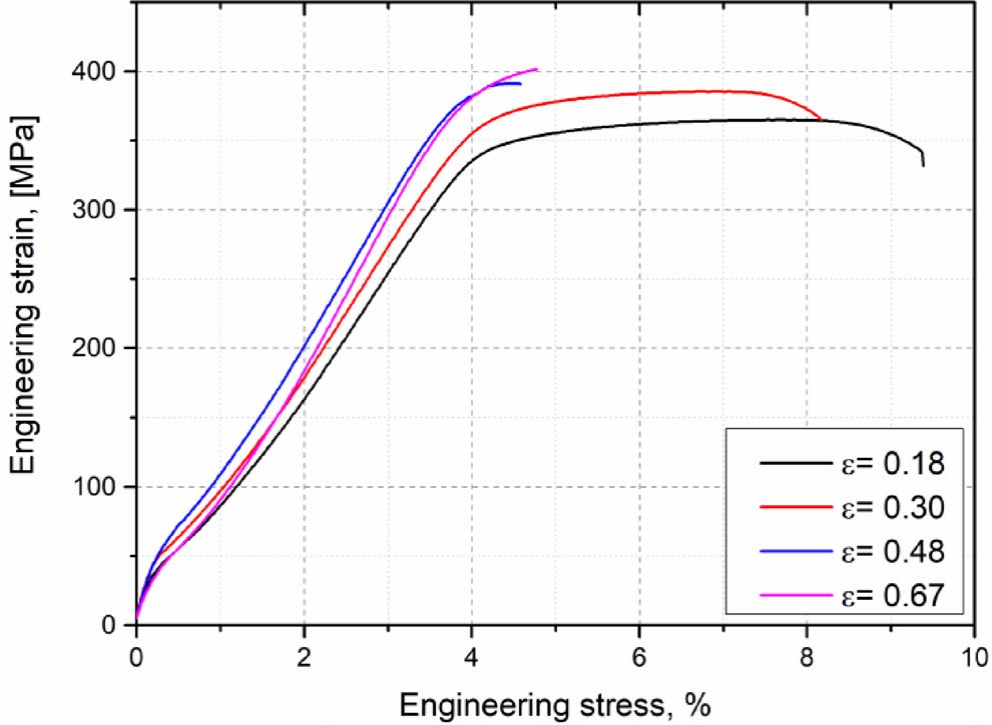
Engineering stress-strain curves of the KOBO-processed AlSi10Mg alloy sample after cold drawing to various strain levels.

Figure 19 shows the true stress-strain curves of the cold-drawn samples. The tensile curves for both as-built and KOBO-processed samples are provided in Supplementary Figure S3. The average values obtained from the tensile test are listed in Table 3.

As revealed, KOBO treatment significantly reduces both the ultimate tensile strength (UTS) and yield strength (YS). Concurrently, the Vickers microhardness also drops substantially (e.g., from 116 HV in the as-built sample to approximately 89 HV post-KOBO, as detailed in Table 3). This overall softening is primarily attributed to dynamic recrystallization (DRX) and the decomposition of the Al-Si cellular network, as separate Si precipitates likely contribute minimally to hardness by acting as weaker obstacles to dislocation movement. However, despite the reduction in strength, KOBO processing remarkably leads to a significantly higher elongation to fracture. This enhanced ductility may be related to the large proportion of boundaries with a misorientation close to the twin boundary, which, as documented by EBSD measurements and literature^48,49^. can arrest crack propagation and provide an effective path for dislocation passage.

Conversely, subsequent cold drawing systematically improves both the UTS and YS, as well as the microhardness, primarily due to dislocation accumulation. For instance, at a drawing strain (ϵ) of 0.18, the UTS increases to 364 MPa (a 12% rise relative to the KOBO-processed sample), and the microhardness rises to 107 HV, though elongation simultaneously drops to 5.7%. Further straining to ϵ=0.30 continues this trend, with UTS reaching 386 MPa and microhardness around 112 HV, while elongation decreases to 4.1%. Notably, after six drawing cycles (ϵ=0.48), the UTS approximates 396 MPa, with hardness reaching 113 HV. Beyond this point, significant further increases in mechanical properties are not observed (e.g., hardness at ϵ=0.63 is 114 HV), and a tensile elongation becomes severely limited to around 0.7% (Table 3).

**Table 3:** Mechanical properties of the tested Samples.

Condition	UTS, MPa	YS, MPa	Elongation, %	Strain hardening exponent, *n*	Hardness, HV
As-built	422 ± 4	185 ± 3	2.4 ± 0.1	0.361	115.8 ± 4.5
KOBO	324 ± 2	255 ± 3	10.2 ± 0.2	0.191	88.4 ± 1.7
Cold-drawn ε = 0.18	364 ± 2	336 ± 2	5.7 ± 0.2	0.167	107.3 ± 1.7
Cold-drawn ε = 0.30	386 ± 3	344 ± 4	4.1 ± 0.1	0.121	112.2 ± 1.6
Cold-drawn ε = 0.48	396 ± 4	352 ± 2	0.7 ± 0.2	*	113.2 ± 3.2
Cold-drawn ε = 0.67	401 ± 4	359 ± 3	0.6 ± 0.3	*	114.6 ± 3.5
* value is statistically unreliable					

**Fig. 20:**
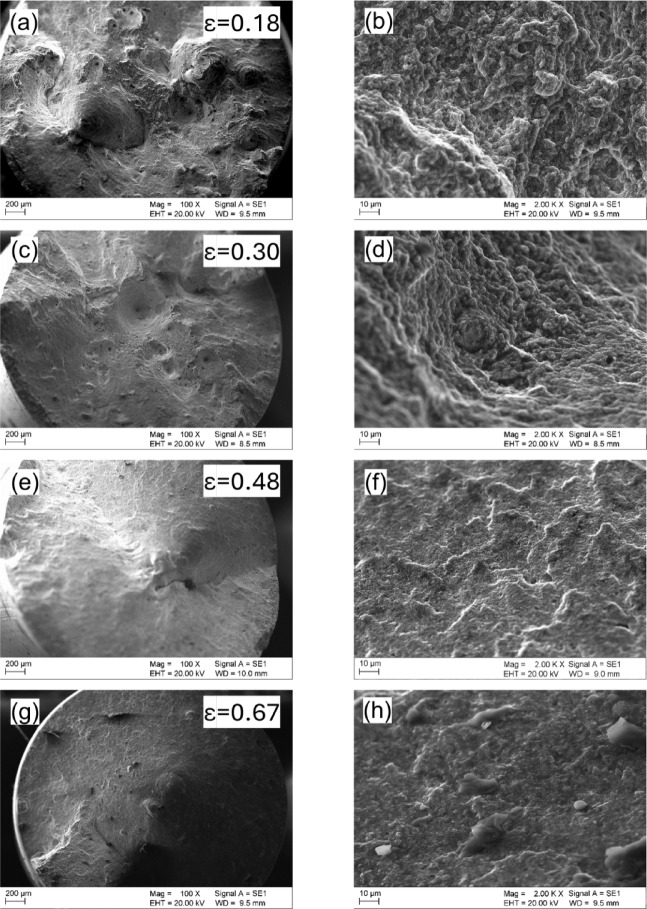
Fracture morphologies of the drawn wire: (**a**) and (**b**) effective strain 0.18, (**c**) and (**d**) effective strain 0.30, (**e**) and (**f**) effective strain 0.48, (**g**) and (**h**) effective strain 0.67.

Figure 20 shows the fracture morphologies of cold-drawn samples. In Fig. 20b, we observe several coarse dimples and microcracks, indicating a more ductile fracture in the sample strained to ε = 0.18. However, upon closer examination in Fig. 20d, we also notice intergranular fracture, revealing that brittle fracture contributes to sample failure as well. As we increase the drawing strain to ε = 0.30, the number and size of dimples decrease, consistent with the observed loss of ductility. The fracture surface of the sample strained to ε = 0.48 mainly consists of cleavage facets, with only a small number of tiny dimples visible in Fig. 20f. As the drawing strain continues to increase, the voids become smaller and shallower, and dimple features nearly disappear. Finally, at ε = 0.67, the fracture morphology is dominated by cleavage facets (Fig. 20h), indicating that the severely deformed specimen fractures in a brittle manner, which is in line with the tensile test results.

## Discussion

This study investigates the microstructural and mechanical evolution of a PBF-LB/M AlSi10Mg alloy subjected to KOBO extrusion and subsequent cold drawing. These combined thermo-mechanical processes transformed the initial as-built microstructure into a refined, ultra-fine-grained (UFG) matrix, reinforced by fragmented silicon particles, creating an in-situ metal matrix composite (MMC)-like architecture. The KOBO-extruded material exhibited a favorable combination of strength and ductility. Subsequent cold drawing systematically tailored these properties, demonstrating a pronounced increase in strength attributable to severe plastic deformation.

The substantial strength enhancement imparted by cold drawing is principally due to extensive grain refinement and the introduction of a high density of crystal defects. Transmission electron microscopy revealed that, in addition to a dense dislocation network, the heavily deformed microstructure contained numerous planar defects, such as stacking faults. These SFs act as formidable barriers to dislocation motion, providing the dominant contribution to the material’s significant strain hardening capacity. The interplay between these defect structures and the overall strengthening response is a central focus of the analysis described below.

The microstructure of the cold-drawn wire, comprising discrete silicon particles embedded within a severely deformed aluminum matrix, permits its mechanical response to be modeled as a composite material. Accordingly, the yield strength of the alloy in the drawn condition can be reasonably estimated using a rule of mixtures approach:

3 $$\:{\sigma\:}_{Al-Si}={f}_{Al}{\sigma\:}_{Al}+{f}_{Si}{\sigma\:}_{Si}$$

in this equation *σ*_Al−Si_ is the calculated alloy yield strength, *σ*_Al_ correspond to the yield strength of aluminum matrix and *σ*_Si_ is the yield strength of Si. The *f*_Al_ and *f*_Si_ are the volume fractions of Al matrix and Si precipitates, respectively. The volume fractions of Al and Si calculated using the lever rule are as follows (*f*_Al_ = 0.77 and *f*_Si_ = 0.23).

Several strengthening components contribute to the yield strength of the Al matrix as follows:

4 $$\:{\sigma\:}_{Al}={\sigma\:}_{0}+{\sigma\:}_{dis}+{\sigma\:}_{gs}+{\sigma\:}_{SS}$$

In the strengthening model σ_0_ the intrinsic lattice friction stress (i.e., the Peierls stress) of the α-Al matrix, for which a value of 10 MPa is assumed. The subsequent terms σ_SS_, σ_dis_, and σ_gs_ denote the individual strengthening contributions arising from solid solution hardening from Si and Mg atoms, the high dislocation density, and grain boundary refinement (the Hall-Petch effect), respectively^50^.

The strength increment arising from increased dislocation density can be expressed as follows:

5 $$\:\varDelta\:{\sigma\:}_{dislocation}=\alpha\:MGb\sqrt{{\rho\:}_{d}}$$

where *α* represents the material constant equal to 0.24, *M* denotes the Taylor factor value equal to 3.06, *G* is the aluminum matrix shear modulus equal to 26 GPa, $\:{\rho\:}_{d}$ is the calculated dislocation density, and *b* is the length of Burgers vector of pure aluminum (~ 0.286 nm). In this study, the dislocation density $\:{\rho\:}_{d}$ value is calculated using the following equation:

6 $$\:{\rho\:}_{d}=\:{\rho\:}_{SSD}+{\rho\:}_{GND}$$

The GNDs density is calculated from EBSD data using the following formula:

7 $$\:{\rho\:}_{GND}=\frac{\alpha\:<{\theta\:}_{ij}>}{b.x}$$

The GNDs density of cold-drawn AlSi10Mg alloy sample is equal to $\:{\rho\:}_{GND}=9.32\times\:{10}^{14}{m}^{-2}$. The SSDs density, calculated from XRD data is equal to $\:{\rho\:}_{SSD}=8.39\times\:{10}^{14}{m}^{-2}$, resulting in a $\:{\rho\:}_{d}=1.77\times\:{10}^{15}{m}^{-2}$. According to Eq. (6), the increased dislocation density contributes to about 212 MPa.

The improved yield strength resulting from the grain refinement is estimated using the Hall-Petch relationship:

7 $$\:\varDelta\:{\sigma\:}_{GB}=\frac{k}{\sqrt{d}}$$

where *k* denotes a Hall-Petch slope (approximately 0.04 MPa·m^1/2^ for Al) and d represents the average grain size estimated from EBSD analysis. Given a grain size of about 0.7 μm, the increase in the yield strength due to grain size strengthening amounts to about 48 MPa.

Typically, dissolved Si atoms cause local lattice distortions, which substantially impede dislocation slip and thereby enhance strength. However, the solubility of Si in aluminum at room temperature is relatively low, at 1.6%^51^. The strength enhancement due to Si in solid solution can be determined with the following equation:

8 $$\:{\varDelta\:\sigma\:}_{Solid\:solution}={K}_{Si}({{\omega\:}_{Si}^{\alpha\:})}^{m}$$

In this equation $\:{K}_{Si}$ the strengthening coefficient for silicon, with a value of 11 MPa wt%^−1^ and $\:{\omega\:}_{Si}^{\alpha\:}$ is the concentration of Si (in weight%) dissolved in the α-Al matrix. The exponent, *m*, is an empirical factor typically ranging from 0.5 to 1. The value for $\:{\omega\:}_{Si}^{\alpha\:}$ was determined using the following modified Vegard’s equation:

9 $$\:\alpha\:=0.40515-0.0174{X}_{Si}$$

In this equation α denotes the aluminum matrix lattice parameter and *X*_*Si*_ is the concentration of Si atoms in the Al matrix. The lattice parameter of aluminum matrix determined using the cos^2^(*θ*)/sin(*θ*) method is equal to 4.0463 Å. By applying Vegard’s law (see Eq. 9), the supersaturation of Si in the Al matrix was calculated to be 2.9 at%. Based on established models for solid solution hardening in Al-Si alloys, this solute concentration corresponds to an increase in yield strength of approximately 33 MPa.

The strengthening contribution from the fragmented silicon particles was modeled using an approach analogous to that employed for the interconnected eutectic network in the as-built condition^52^. This load-transfer model, a form of the rule of mixtures, considers the Si particles as a reinforcing phase within the aluminum matrix. Assuming a silicon volume fraction of 23% and an intrinsic yield strength of the Si phase 380 MPa, the contribution of the particles to the overall strength of the composite is calculated to be 87 MPa.

Considering the above calculations, the overall yield strength of the cold-drawn wire can be expressed as follows:

10 $$\:{\sigma\:}_{Al-Si}=0.77\times\:303\:MPa+0.23\times\:380\:MPa$$

11 $$\:{\sigma\:}_{Al-Si}=321\:MPa$$

The estimated YS value of 321 MPa is lower than the YS value derived from the tensile tests. The observed deviation could be because the contribution of stacking fault/twin boundary strengthening was not considered in the proposed model. Notably, transmission electron microscopy (TEM) analysis reveals the presence of stacking faults with an average length of 8 nm within the aluminum matrix. These planar defects play a crucial role in the dynamic Hall-Petch effect. Specifically, stacking faults act as obstacles to moving dislocations, leading to their accumulation and ultimately contributing to the overall improvement in yield strength^53^. In several recent studies, researchers have documented the significant contribution of stacking faults to the overall yield strength of the aluminum alloys. For example, Zhou et al.^54^ revealed that SFs contributed significantly to the enhancement of the yield strength of Al(Mg)-Al_3_Mg_2_ composite, accounting for ~ 36% of the total yield strength. In another study, Zhang et al.^55^ who studied the microstructure of the nanograined Al-Mg-Y alloy showed that the SFs and 9R phase contributed to 100 MPa, increasing the hardness of the alloy by ~ 9%.

The contribution of stacking faults to the alloy yield strength is often expressed using the following formula^56^:

12 $$\:\varDelta\:{\sigma\:}_{SF}=\frac{{K}_{SF}}{{L}_{SF}}$$

Where $\:{K}_{SF}$ is material-depended constant, usually assumed as 5000 nm*MPa for aluminum alloys. Considering the calculated mean spacings between SFs in the cold-drawn sample $\:{L}_{SF}$=~27 nm, and the fraction of aluminum phase of 77%, Eq. (12) yield the value of $\:\varDelta\:{\sigma\:}_{SF}$=142 MPa. This value is much larger than the difference between the calculated and measured yield strength (~ 31 MPa).

An additional, unquantified contribution to the alloy’s yield strength likely arises from point defects, specifically self-interstitial atoms (SIAs), which were not included in the model. The formation of a high density of SIAs is a known characteristic of materials subjected to severe plastic deformation via the KOBO process^30,57^. These point defects and their small clusters act as potent obstacles to dislocation motion, thereby increasing the yield strength and influencing strain hardening behavior^58^. Although SIAs can contribute significantly to strengthening, their direct observation and quantification by conventional TEM is notoriously difficult, making their precise contribution challenging to model.

**Fig. 21:**
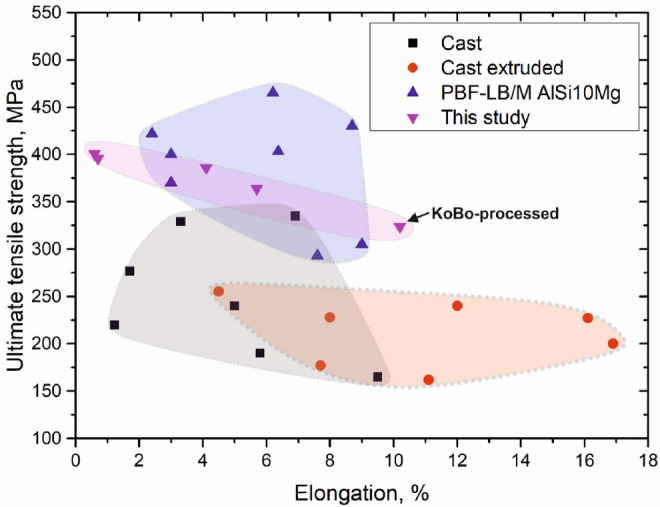
Tensile stress versus elongation plot for various Al-Si alloys, including data from this work and literature^18,59–75^.

Based on the above analyses, it is concluded that the dislocations and second phase particles play the dominant role in the strengthening of cold-drawn KOBO-processed PBF-LB/M AlSi10Mg alloy. Considering the complexity of the proposed manufacturing method, it has become imperative to compare the mechanical properties of cold-drawn wire with the data available in the literature for Al-Si alloys, as shown in Fig. 21.

In summary, the KOBO-extruded PBF-LB/M AlSi10Mg alloy demonstrates superior mechanical performance, specifically, higher tensile strength, than its cast and cast-extruded Al-Si counterparts. This suggests that KOBO extrusion holds significant promises for tailoring the performance of PBF-LB/M components. The strength is further enhanced by multi-pass cold drawing, which promotes dislocation accumulation and results in a UTS of $\:\sim$401 MPa. However, this comes at the cost of reduced ductility. While this brittleness limits structural use, the processing route explored in this work provides a pathway to producing components suitable for applications where minimal deformation is expected, such as aerospace fasteners or tension elements. Mitigation strategies such as inter-pass annealing to partially recover ductility without sacrificing strength^76^, could be explored in future work. Although Fig. 21 benchmarks the alloy against cast/extruded references, the processing route also offers advantages over other post-AM treatments like hot forging or hybrid AM approaches by producing fully transformed, refined microstructure without relying on high-temperature treatments.

## Conclusions

In this study, an AlSi10Mg alloy, produced PBF-LB/M underwent a multi-step plastic deformation post-processing procedure involving KOBO extrusion followed by cold drawing to fabricate a high-strength wire. Key findings from the study are summarized below:

1. KOBO extrusion facilitated the refinement of the Al-Si cellular structure, resulting in a more uniform dispersion of Si particles within the Al-matrix. In addition to particle refinement, the KOBO extrusion resulted in a significant refinement of PBF-LB/M AlSi10Mg alloy grain microstructure.
2. Subsequent cold drawing refined the grain structure by approximately 20%. A large density of dislocations piled up during cold drawing leading to the formation of dense dislocation walls (DDWs) and resulting in the formation of sub-grain boundaries. The results suggest a multiple grain refinement mechanism involving formation of dense dislocation structures and SFs intersection.
3. After cold drawing most of the twin boundaries have lost their twin relationships due to the grain rotations during the multi-stage plastic deformation.
4. The yield strength and hardness of the cold-drawn wire increased with greater cold-drawing strain. This behavior was significantly influenced by the presence of uniformly distributed Si particles within the aluminum matrix, as well as by the accumulation of dislocations. Experimental results show that the Si phase particles contributed approximately 24%, while the dislocations accounted for about 34% of the overall yield strength.

## Supplementary Information

Below is the link to the electronic supplementary material.

Supplementary Material 1

## Data Availability

The datasets used and/or analysed during the current study available from the corresponding author on reasonable request.
