# Supplementary material for: Utilizing PBF-LB/M AlSI10Mg alloy post-processed via KOBO-extrusion and subsequent cold drawing to obtain high-strength wire
Source: Sci Rep. 2025 Aug 23;15:31025. doi: 10.1038/s41598-025-14980-3 (PMC12375056; doi:10.1038/s41598-025-14980-3)
Supplement: Supplementary file 1 — Supplementary Material 1 [file 41598_2025_14980_MOESM1_ESM.docx]

Supplementary material associated with article entitled:

**Utilizing PBF-LB/M AlSI10Mg alloy post-processed via KOBO-extrusion and subsequent cold drawing to obtain high-strength wire**

P. Snopiński^1,*^, A. Appiah^2^, K. Matus^2^, Ł. Kuczek^3^, K.Żaba^3^, M. Balcerzak^3^, J. Hajnyš^4^

^1^Department of Engineering Materials and Biomaterials, Silesian University of Technology, 18A Konarskiego Street, 44-100 Gliwice, Poland

^2^Materials Research Laboratory, Faculty of Mechanical Engineering, Silesian University of Technology, 18A Konarskiego Street, 44-100 Gliwice, Poland

^3^Department of Metal Working and Physical Metallurgy of Non-Ferrous Metals, AGH University of Science and Technology, Al. Adama Mickiewcza 30, 30-059 Cracow, Poland

^4^Faculty of Mechanical Engineering, VSB-TU Ostrava, 17. Listopadu 2172/15, Ostrava 708 00, Czech Republic


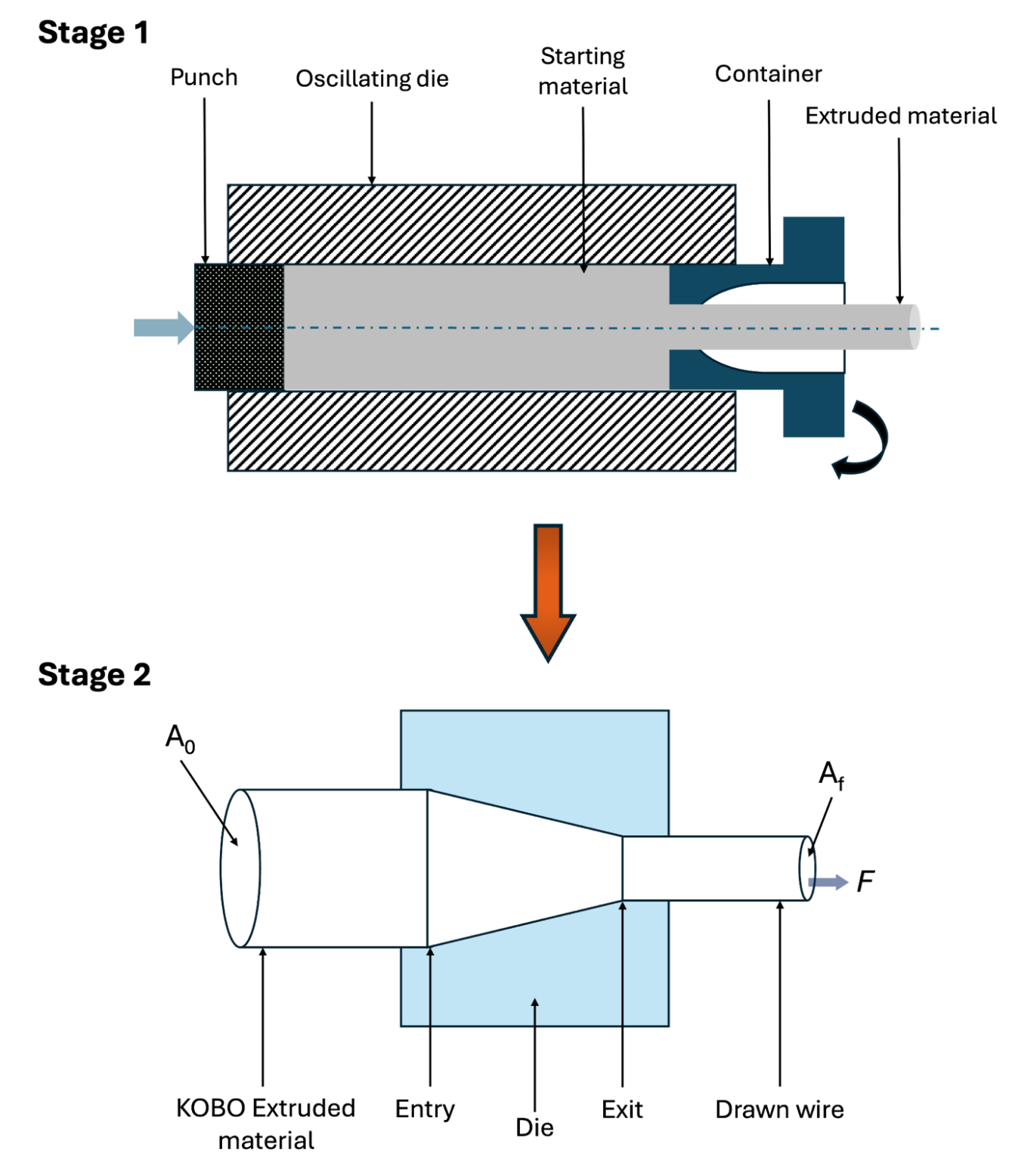


Fig. S1. Schematic diagram of the wire fabrication process following PBF-LB/M alloy processing. Stage 1. KOBO extrusion, and Stage 2. Cold drawing

| a)    1 | b)   |
| --- | --- |
|  | c)  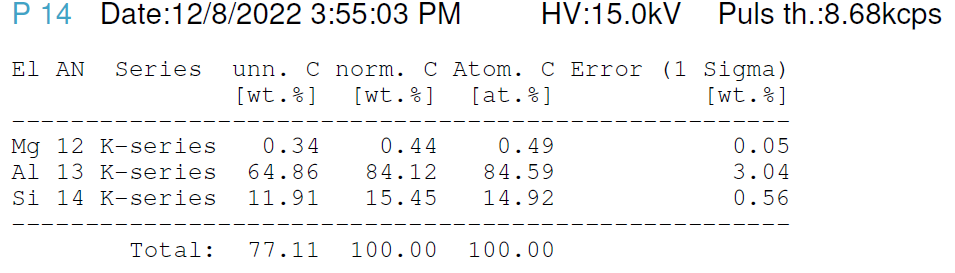 |

Fig. S2. Energy-Dispersive X-ray Spectroscopy (EDS) analysis of the KOBO-processed sample. (a) SEM micrograph showing the area of analysis. (b) EDS spectrum acquired from the point indicated as "1" in (a). (c) Table summarizing the elemental composition (in wt.% and at.%) from the point analysis.

| a) |
| --- |
| b) |

Fig. S3. Engineering stress vs engineering strain curves a) as-built, b) KOBO-processed AlSi10Mg alloy sample
